# Early-Life Exposure to Air Pollution and Childhood Asthma Cumulative Incidence in the ECHO CREW Consortium

**DOI:** 10.1001/jamanetworkopen.2024.0535

**Published:** 2024-02-28

**Authors:** Antonella Zanobetti, Patrick H. Ryan, Brent A. Coull, Heike Luttmann-Gibson, Soma Datta, Jeffrey Blossom, Cole Brokamp, Nathan Lothrop, Rachel L. Miller, Paloma I. Beamer, Cynthia M. Visness, Howard Andrews, Leonard B. Bacharier, Tina Hartert, Christine C. Johnson, Dennis R. Ownby, Gurjit K. Khurana Hershey, Christine L.M. Joseph, Eneida A. Mendonça, Daniel J. Jackson, Edward M. Zoratti, Anne L. Wright, Fernando D. Martinez, Christine M. Seroogy, Sima K. Ramratnam, Agustin Calatroni, James E. Gern, Diane R. Gold, For the ECHO Children’s Respiratory and Environmental Workgroup

**Affiliations:** 1 Department of Environmental Health, Harvard T.H. Chan School of Public Health, Boston, Massachusetts; 2 Department of Pediatrics, University of Cincinnati, College of Medicine, Cincinnati, Ohio; 3 Division of Biostatistics and Epidemiology, Cincinnati Children’s Hospital Medical Center, Cincinnati, Ohio; 4 Department of Biostatistics, Harvard T.H. Chan School of Public Health, Boston, Massachusetts; 5 Channing Division of Network Medicine, Brigham and Women’s Hospital and Harvard Medical School, Boston, Massachusetts; 6 Center for Geographic Analysis, Harvard University, Cambridge, Massachusetts; 7 Asthma and Airways Disease Research Center, University of Arizona, Tucson; 8 Department of Community, Environment, and Policy, Mel and Enid Zuckerman College of Public Health, University of Arizona, Tucson; 9 Division of Clinical Immunology, Icahn School of Medicine at Mount Sinai, New York, New York; 10 Rho Inc, Federal Research Operations, Durham, North Carolina; 11 Department of Biostatistics, Mailman School of Public Health, Columbia University, New York, New York; 12 Monroe Carell Jr Children’s Hospital at Vanderbilt, Division of Pediatric Allergy, Immunology, and Pulmonary Medicine, Nashville, Tennessee; 13 Vanderbilt University School of Medicine, Division of Allergy, Pulmonary, and Critical Care Medicine, Nashville, Tennessee; 14 Department of Public Health Sciences, Henry Ford Health, Detroit, Michigan; 15 Division of Allergy and Immunology, Augusta University, Augusta, Georgia; 16 Cincinnati Children’s Hospital, Division of Asthma Research, Cincinnati, Ohio; 17 Department of Pediatrics, Indiana University, Indianapolis; 18 Department of Pediatrics, University of Wisconsin School of Medicine and Public Health, Madison; 19 Department of Medicine, Henry Ford Health, Detroit, Michigan; 20 Division of Pulmonary and Sleep Medicine, Department of Pediatrics, College of Medicine, University of Arizona, Tucson

## Abstract

**Importance:**

Exposure to outdoor air pollution contributes to childhood asthma development, but many studies lack the geographic, racial and ethnic, and socioeconomic diversity to evaluate susceptibility by individual-level and community-level contextual factors.

**Objective:**

To examine early life exposure to fine particulate matter (PM_2.5_) and nitrogen oxide (NO_2_) air pollution and asthma risk by early and middle childhood, and whether individual and community-level characteristics modify associations between air pollution exposure and asthma.

**Design, Setting, and Participants:**

This cohort study included children enrolled in cohorts participating in the Children’s Respiratory and Environmental Workgroup consortium. The birth cohorts were located throughout the US, recruited between 1987 and 2007, and followed up through age 11 years. The survival analysis was adjusted for mother’s education, parental asthma, smoking during pregnancy, child’s race and ethnicity, sex, neighborhood characteristics, and cohort. Statistical analysis was performed from February 2022 to December 2023.

**Exposure:**

Early-life exposures to PM_2.5_ and NO_2_ according to participants’ birth address.

**Main Outcomes and Measures:**

Caregiver report of physician-diagnosed asthma through early (age 4 years) and middle (age 11 years) childhood.

**Results:**

Among 5279 children included, 1659 (31.4%) were Black, 835 (15.8%) were Hispanic, 2555 (48.4%) where White, and 229 (4.3%) were other race or ethnicity; 2721 (51.5%) were male and 2596 (49.2%) were female; 1305 children (24.7%) had asthma by 11 years of age and 954 (18.1%) had asthma by 4 years of age. Mean values of pollutants over the first 3 years of life were associated with asthma incidence. A 1 IQR increase in NO_2_ (6.1 μg/m^3^) was associated with increased asthma incidence among children younger than 5 years (HR, 1.25 [95% CI, 1.03-1.52]) and children younger than 11 years (HR, 1.22 [95% CI, 1.04-1.44]). A 1 IQR increase in PM_2.5_ (3.4 μg/m^3^) was associated with increased asthma incidence among children younger than 5 years (HR, 1.31 [95% CI, 1.04-1.66]) and children younger than 11 years (OR, 1.23 [95% CI, 1.01-1.50]). Associations of PM_2.5_ or NO_2_ with asthma were increased when mothers had less than a high school diploma, among Black children, in communities with fewer child opportunities, and in census tracts with higher percentage Black population and population density; for example, there was a significantly higher association between PM_2.5 _and asthma incidence by younger than 5 years of age in Black children (HR, 1.60 [95% CI, 1.15-2.22]) compared with White children (HR, 1.17 [95% CI, 0.90-1.52]).

**Conclusions and Relevance:**

In this cohort study, early life air pollution was associated with increased asthma incidence by early and middle childhood, with higher risk among minoritized families living in urban communities characterized by fewer opportunities and resources and multiple environmental coexposures. Reducing asthma risk in the US requires air pollution regulation and reduction combined with greater environmental, educational, and health equity at the community level.

## Key Points

**Question:**

Is there an association between early life exposure to air pollution and the risk of asthma by early and middle childhood, and is this association modified by individual and community-level characteristics?

**Findings:**

In this cohort study of 5279 children, mean fine particulate matter (PM_2.5_) and mean nitrogen dioxide (NO_2_) air pollution during the first 3 years of life were associated both with asthma incidence by early and by middle childhood, after adjusting for individual-level characteristics. The association of ambient pollution (PM_2.5_ or NO_2_) with incident asthma was modified by community-level and individual-level socioeconomic circumstances, including maternal education and race.

**Meaning:**

These findings suggest that exposure to PM_2.5_ or NO_2_ air pollution during early childhood may play a role in the development of childhood asthma, with higher risk among minoritized families living in densely populated communities characterized by fewer opportunities and resources and multiple environmental coexposures.

## Teaser

This cohort study examines the association of exposure to air pollution with asthma incidence in early and middle childhood and investigates whether individual and community-level characteristics modify this association.

## Introduction

Air pollution is a near ubiquitous exposure and the largest environmental contributor to disease and premature death in the world, including for children.^1,2^ Exposure to air pollution has been consistently associated with respiratory morbidity, including wheeze and exacerbation of asthma in children. Several reviews^3,4,5,6^ on air pollution, asthma, and respiratory symptoms in children concluded that outdoor traffic pollution contributes to the development of childhood asthma.

However, community-level and individual-level contextual factors that increase not only exposure, but also susceptibility to air pollution–related childhood asthma effects, including the developmental stage and age of onset of asthma, remain poorly understood. Most prior cohort studies with individual-level information lacked the geographic, racial and ethnic, and socioeconomic diversity to explore the modifying role of community-level contextual factors and the association between air pollution exposure and asthma development.

The Environmental Influences on Child Health Outcomes (ECHO) Children’s Respiratory and Environmental Workgroup (CREW)^7^ consortium, a US nationwide birth cohorts network, is well-positioned to address these questions given the multiple decades of recruitment and follow-up of birth cohorts, the geographic and demographic diversity of study participants,^8^ the wide distribution of state-of-the-art spatio-temporally-resolved fine particulate matter (PM_2.5_) and nitrogen dioxide (NO_2_) estimates, and the diverse individual and area-level socioeconomic and built environment factors that vary across and within cohorts.

Previously, we found in the CREW consortium that Black and Hispanic children and children who resided in census tracts with higher rates of household poverty and population density were at increased risk for developing childhood asthma.^9^ Building upon these findings, we hypothesized that (1) early life exposure to PM_2.5_ and NO_2_ is associated with increased asthma risk by early and middle childhood, and (2) not only individual-level, but also community-level factors (eg, living in areas with higher level of poverty, and less opportunity) are associated with increased child susceptibility to pollution effects on asthma risk.

## Methods

### Study Population

Our study population included 8 of the 12 longitudinal birth cohorts participating in the Children’s Respiratory and Environmental Workgroup (CREW), representing a diverse sample of children and their families residing throughout the US in urban, suburban, and rural environments (eMethods 1 and eTable 1 in Supplement 1). We excluded cohorts with young children without harmonized asthma outcomes, and we excluded our oldest cohort for which early-life air pollution estimates were not available.

Eligibility criteria, study recruitment, and other methods, have been previously described.^7^ Birth years of cohort participants spanned from 1987 to 2007, and participants were followed up to age 11. All cohorts had institutional review board approvals from each participating cohort’s institution and all participants provided written informed consent. This cohort study followed the Strengthening the Reporting of Observational Studies in Epidemiology (STROBE) reporting guideline.

### Health Outcomes

We defined asthma as caregiver report of physician-diagnosed asthma. We ascertained the outcomes through survival analysis as asthma incidence by less than 5 years of age (early childhood) and by less than 12 years of age (middle childhood); and, secondarily, through logistic regression as ever asthma through age 4 years and through age 11 years. As a sensitivity analysis, we also considered ever asthma before age 4 years with any wheeze reported after age 3 years, to ascertain whether associations were consistent for those children diagnosed before age 4 years who had an indication of persistent (rather than transient) asthma.

### Individual Characteristics

Cohorts provided individual characteristics, including child’s sex, caregiver-reported child race and ethnicity (Hispanic, non-Hispanic Black [Black], non-Hispanic White [White], and other [for any race or ethnicity not in the preceding categories]), mother’s education (no high school diploma, high school diploma, and college and graduate school), maternal-reported smoking during pregnancy, and parental history of asthma defined as history in the mother, father, or both vs no parental history. We acknowledge that race is a social construct and correlates with poverty, adverse physical environments, unequal access to health care, and a multitude of structural, systemic, and institutional determinants. The CREW Biostatistics/Bioinformatics Core collected and harmonized data from each cohort.

### Exposure Assessment: PM_2.5_ and NO_2_

We obtained daily estimates of PM_2.5_ and NO_2_ from 2000 to 2016 at a 1 km^2^ resolution from previously validated prediction models^10,11,12^ and monthly PM_2.5_ predictions at the 6 km^2^ grid for the years 1988-2007 from a previously published model^12^ to estimate PM_2.5_ and NO_2_ exposures (eMethods 2 in Supplement 1).

The 1 km^2^ estimates were linked to the home addresses for each CREW participant using our Decentralized Geomarker Assessment for Multi-site Studies (DeGAUSS) software^13,14^ approach (eMethods 2 in Supplement 1). For the cohorts with person-time before 2000 when the 1 km^2^ estimates were not available, we created cohort-specific calibration factors based on monthly mean values, using all available overlap data to obtain calibrated annual, monthly, and prenatal 6 km^2^ exposure estimates for periods before 2000 (eMethods 2 in Supplement 1).

Annual mean values for each year of life beginning at the birth date through age 1 year and up to age 5 years were calculated. In addition, we calculated mean values from birth through age 1 year, 1 to 2 years, 1 to 3 years, and 1 to 4 years. As NO_2_ estimates were only available from 2000 and later, we were not able to assign early life NO_2_ exposures to 2 cohorts (Children’s Asthma Study, Epidemiology of Home Allergens and Asthma Study), whose children enrolled at birth in the 1990s.

### Neighborhood-Level Characteristics

We obtained US census data for percentage population with low income, percentage Black population, population density, median household income, and percentage low-income families. We also obtained the Child Opportunity Index (COI),^15,16^ a measure of the quality of neighborhoods in which children live, and the social vulnerability index (SVI),^17^ a measure used to identify high-risk populations that are especially at risk during public health emergencies. COI is a score based on neighborhood-level indicators, grouped into 3 domains, with higher scores reflecting more favorable neighborhood opportunities. SVI is computed from census variables as percentile ranging from 0 (lowest risk) to 1 (highest risk), grouped into 4 domains (see eMethods 2, eFigure, 1, and eFigure 2 in Supplement 1).

### Statistical Analysis

#### Primary Analysis

We analyzed the association between exposures and asthma incidence with a Cox proportional hazard model, adjusting for potential confounders including mother’s education, child’s race and ethnicity, sex, smoking during pregnancy, parental history of asthma and an indicator variable for cohort. In secondary analysis, we examined the association between exposure and ever asthma using logistic regression models, adjusting for the same confounders as in the survival analysis.

We examined effect modification by child’s race and ethnicity and sex, mother’s education, and neighborhood socioeconomic factors, including an interaction term between each pollutant and each modifier in separate models. We then computed the association of pollution in each category of the modifier. For continuous modifiers (neighborhood socioeconomic factors), we computed the association of pollution at the 10th (Low) and 90th (High) percentiles of the modifier. We reported the results as hazard ratios (HRs) for the survival analysis and odds ratios (ORs) for the logistic regression with 95% CI for a 1 IQR increase in each pollutant. *P* < .05 was considered as the significance level. Statistical analysis was performed using R version 4.4.1 (R Project for Statistical Computing) and SAS version 9.4 (SAS Institute) from February 2022 to December 2023.

#### Sensitivity Analyses

We repeated the analysis using asthma incidence by less than 5 years of age with any wheeze reported after age 3 years. To account for potential residual spatial correlation, we repeated the analysis using mixed-effect models adding random intercepts for census tract, in addition to the indicator variable for cohort. We then applied a multinomial regression, where the outcome was a categorical variable defined as asthma by age 4 years and first asthma diagnosis between ages 5 and 11 years.

## Results

Among a total of 5279 children included, 1659 (31.4%) were Black, 835 (15.8%) were Hispanic, 2555 (48.4%) where White, and 229 (4.3%) were other race or ethnicity; 2721 (51.5%) were male and 2596 (49.2%) were female; 1305 children (24.7%) had asthma by 11 years of age and 954 (18.1%) had asthma by 4 years of age; 3315 mothers (62.8%) had some college or higher education, 565 mothers (10.7%) smoked during pregnancy, and 1893 children (35.9%) had parents with history of asthma. Table 1 presents these characteristics for each CREW cohort. eFigure 3 in Supplement 1 shows the flowchart of the analytic data set. eTables 2, 3, 4, and 5 in Supplement 1 present the characteristics when asthma, PM_2.5_, and NO_2_ are missing. The distribution of the outcomes and the individual characteristics varied widely across cohorts, demonstrating racial and socioeconomic diversity among participants. The Figure shows the distributions of PM_2.5_ and NO_2_ as well as the neighborhood-level variables; all of the variables present substantial variability across and within cohorts. eFigure 4 in Supplement 1 shows the correlation among the neighborhood-level variables and the pollutants. PM_2.5_ was not correlated with any variables, whereas NO_2_ correlated with population density (*r* = 0.63). The census variables were positively correlated with SVI (eg, correlation between total SVI and percentage of Black population: *r* = 0.63) and negatively correlated with COI (eg, correlation between total COI and percentage of Black population: *r* = −0.64).

**Table 1.** Child and Caregiver Demographic Characteristics and Distribution of Respiratory Health Outcomes Among CREW Participants

	No. (%)											
	CAS	CCAAPS	CCCEH	COAST	EHAAS	IIS	URECA Baltimore	URECA Boston	URECA New York	URECA St Louis	WHEALS	Total
Child sex												
Male	377 (50.1)	415 (54.5)	346 (48.7)	160 (58.0)	260 (53.4)	231 (50.5)	85 (52.1)	74 (53.6)	64 (59.3)	86 (48.3)	623 (49.9)	2721 (51.5)
Female	386 (51.3)	347 (45.5)	364 (51.3)	120 (43.5)	227 (46.6)	235 (51.4)	78 (47.9)	66 (47.8)	52 (48.1)	92 (51.7)	629 (50.4)	2596 (49.2)
Child race and ethnicity												
Hispanic	20 (2.7)	10 (1.3)	461 (64.9)	8 (2.9)	37 (7.6)	115 (25.2)	4 (2.5)	40 (29.0)	65 (60.2)	4 (2.2)	71 (5.7)	835 (15.8)
Non-Hispanic Black	12 (1.6)	147 (19.3)	249 (35.1)	12 (4.3)	33 (6.8)	8 (1.8)	146 (89.6)	76 (55.1)	37 (34.3)	160 (89.9)	779 (62.4)	1659 (31.4)
Non-Hispanic White	686 (91.2)	560 (73.5)	0 (0.0)	251 (90.9)	387 (79.5)	305 (66.7)	5 (3.1)	8 (5.8)	0 (0.0)	8 (4.5)	345 (27.6)	2555 (48.4)
Other^a^	34 (4.5)	45 (5.9)	0 (0.0)	5 (1.8)	30 (6.2)	28 (6.1)	8 (4.9)	14 (10.1)	6 (5.6)	6 (3.4)	53 (4.2)	229 (4.3)
Mother’s education												
College and graduate school	470 (62.5)	557 (73.1)	168 (23.7)	250 (90.6)	452 (92.8)	362 (79.2)	12 (7.4)	30 (21.7)	29 (26.9)	38 (21.3)	947 (75.9)	3315 (62.8)
High school	258 (34.3)	136 (17.8)	280 (39.4)	19 (6.9)	29 (6.0)	65 (14.2)	53 (32.5)	36 (26.1)	13 (12.0)	45 (25.3)	227 (18.2)	1161 (22.0)
No high school	24 (3.2)	49 (6.4)	251 (35.4)	4 (1.4)	6 (1.2)	28 (6.1)	98 (60.1)	72 (52.2)	66 (61.1)	95 (53.4)	74 (5.9)	767 (14.5)
Parental history of asthma	106 (14.1)	266 (34.9)	196 (27.6)	179 (64.9)	250 (51.3)	143 (31.3)	99 (60.7)	95 (68.8)	81 (75.0)	101 (56.7)	377 (30.2)	1893 (35.9)
Smoking during pregnancy	123 (16.4)	90 (11.8)	14 (2.0)	12 (4.3)	30 (6.2)	47 (10.3)	30 (18.4)	26 (18.8)	7 (6.5)	38 (21.3)	148 (11.9)	565 (10.7)
Asthma by age 11 y	122 (16.2)	132 (17.3)	273 (38.5)	109 (39.5)	163 (33.5)	73 (16.0)	55 (33.7)	53 (38.4)	43 (39.8)	85 (47.8)	197 (15.8)	1305 (24.7)
Asthma by age 4 y	68 (9.0)	83 (10.9)	189 (26.6)	48 (17.4)	125 (25.7)	58 (12.7)	48 (29.4)	48 (34.8)	38 (35.2)	69 (38.8)	180 (14.4)	954 (18.1)
Duration of follow-up, mean (SD), y	9 (3.3)	6 (1.8)	6 (4.0)	8 (3.4)	8 (4.2)	7 (3.9)	7 (4.3)	6 (4.6)	7 (4.5)	6 (4.3)	6 (3.9)	7.3 (3.8)
Children, total No.	752	762	710	276	487	457	163	138	108	178	1248	5279

Abbreviations: CAS, Children’s Asthma Study; CCAAPS, Cincinnati Childhood Allergy and Air Pollution Study; CCCEH, Columbia Center for Children’s Environmental Health Cohort; COAST, Childhood Origins of Asthma Study; CREW, Children’s Respiratory and Environmental Workgroup; EHAAS, Epidemiology of Home Allergens and Asthma Study; IIS, Infant Immune Study; URECA, Urban Environment and Childhood Asthma Study; WHEALS, Wayne County Health Environment Allergy and Asthma Longitudinal Study.

^a^ The other race and ethnicity category included any race or ethnicity not categorized as Hispanic, non-Hispanic Black, or non-Hispanic White.

**Figure.**
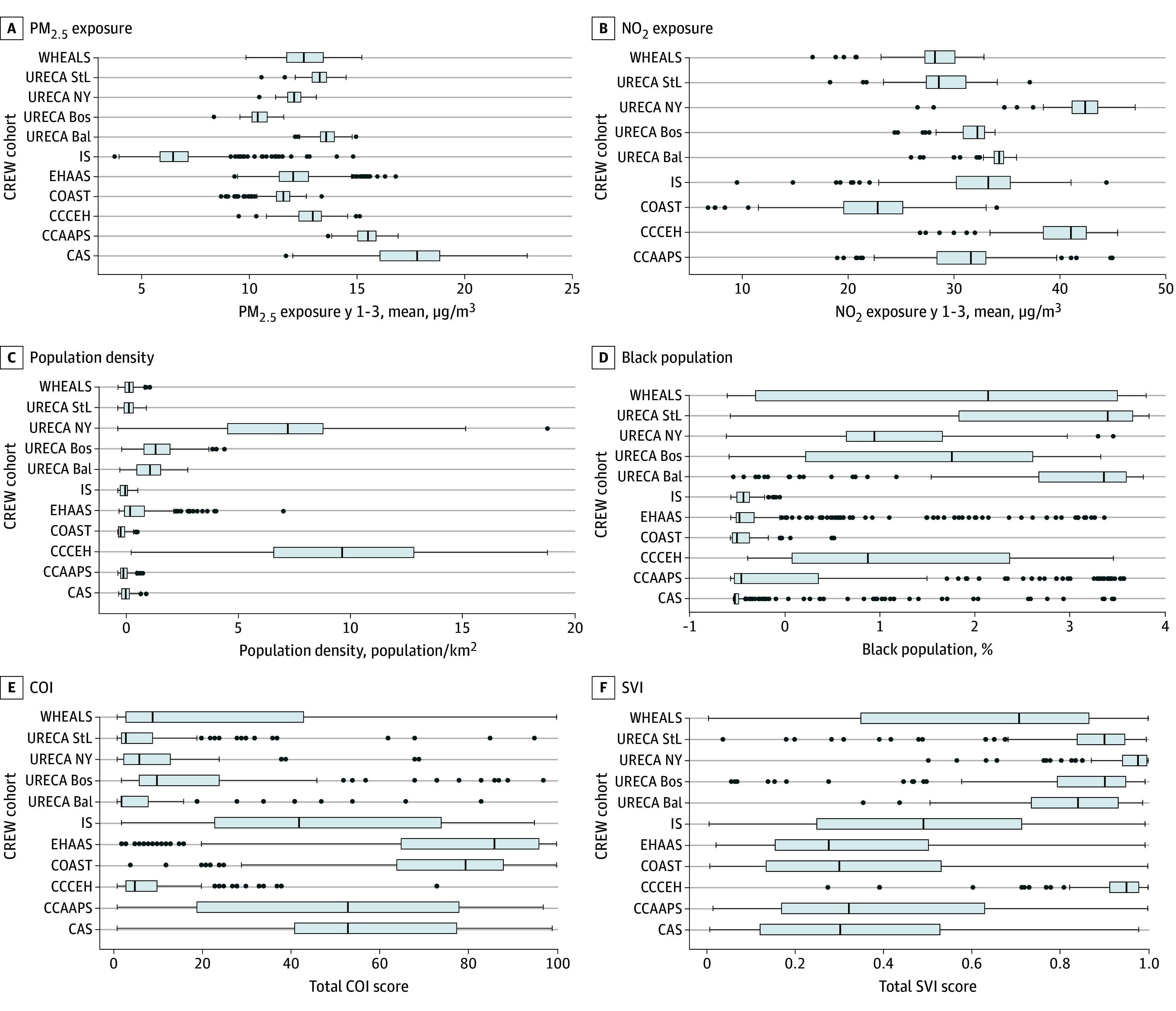
Distributions of the Exposures, US Census Variables of Percentage Black Population and Population Density, and Child Opportunity Index (COI) and Social Vulnerability Index (SVI) Across the Children’s Respiratory and Environmental Workgroup (CREW) Cohorts The figures shows cohort-specific boxplots, where the box is used to represent the IQR, or the data between the first and third quartile, the line within the box represents the median, the whiskers extend from each quartile to the minimum and maximum values, and the points beyond the whiskers represents the outliers. Bal indicates Baltimore; Bos, Boston; CAS, Children’s Asthma Study; CCAAPS, Cincinnati Childhood Allergy and Air Pollution Study; CCCEH, Columbia Center for Children’s Environmental Health Cohort; COAST, Childhood Origins of Asthma Study; COI, Child Opportunity Index; EHAAS, Epidemiology of Home Allergens and Asthma Study; IIS, Infant Immune Study; NO_2_, nitrogen dioxide air pollution; NY, New York; PM_2.5_, fine particulate matter air pollution; StL, St Louis; SVI, Social Vulnerability Index; URECA, Urban Environment and Childhood Asthma Study; WHEALS, Wayne County Health Environment Allergy and Asthma Longitudinal Study.

Table 2 and eFigure 5 in Supplement 1 present the results of the association between early life air pollution exposure and asthma incidence by younger than 5 and younger than 12 years of age using survival analysis and logistic regression. We found stronger associations with both outcomes and pollutants averaged over the first 2 and 3 years of life compared with other pollution averages (eFigure 5 in Supplement 1). Specifically, we found that 1 IQR increase in mean NO_2_ (6.1 μg/m^3^) over the first 3 years of life was associated with increased asthma incidence through the first 4 years of life (HR, 1.25 [95% CI, 1.03-1.52]) and through the first 11 years of life (HR, 1.22 [95% CI, 1.04-1.44]). For the same 3-year average, a 1 IQR increase in mean PM_2.5_ (3.4 μg/m^3^) was associated with increased asthma incidence among children younger than 5 years (HR, 1.31 [95% CI, 1.04-1.66]) and children younger than 12 years (HR, 1.23 [95% CI, 1.01-1.507]). NO_2_ and PM_2.5_ were also significantly associated with increased odds of asthma through age 4 years and through age 11 years.

**Table 2.** Risk of Asthma Incidence and Odds of Asthma for PM_2.5_ and NO_2_ for Early Years of Life

	4 y		11 y	
	NO_2_	PM_2.5_	NO_2_	PM_2.5_
Survival analysis, HR (95% CI)^a^				
First y of life	1.10 (0.93-1.30)	1.25 (1.01-1.53)	1.13 (0.98-1.30)	1.15 (0.97-1.37)
Mean of y 1-2	1.23 (1.03-1.47)	1.27 (1.03-1.58)	1.19 (1.03-1.39)	1.19 (0.99-1.42)
Mean of y 1-3	1.25 (1.03-1.52)	1.31 (1.04-1.66)	1.22 (1.04-1.44)	1.23 (1.01-1.50)
**Logistic regression, OR (95% CI)** ^b^				
First y of life	1.15 (0.94-1.40)	1.26 (1.00-1.59)	1.23 (1.02-1.47)	1.18 (0.97-1.46)
Mean of y 1-2	1.31 (1.07-1.62)	1.32 (1.03-1.69)	1.30 (1.08-1.57)	1.25 (1.01-1.54)
Mean of y 1-3	1.31 (1.05-1.64)	1.39 (1.06-1.81)	1.31 (1.07-1.60)	1.30 1.03-1.65)

Abbreviations: HR, hazard ratio; NO_2_, nitrogen dioxide air pollution; OR, odds ratio; PM_2.5_, fine particulate matter air pollution.

^a^ The survival analysis assessed asthma incidence though the first 4 and 11 years of life.

^b^ The logistic regression assessed odds of asthma through age 4 and 11 years.

Table 3 presents results of air pollution modified by individual characteristics and by a selection of neighborhood-level variables separately for asthma incidence through the first 4 and 11 years of life. We found that the associations were stronger when mothers had less than a high school diploma and among Black children; for example, there was a significantly higher association between PM_2.5 _and asthma incidence by less than 5 years of age in Black children (HR, 1.60 [95% CI, 1.15-2.22]) compared with White children (HR, 1.17 [95% CI, 0.90-1.52]) (Table 3).

**Table 3.** PM_2.5_ and NO_2_ by Individual Characteristics and Selected Neighborhood Characteristics^a^

	HR (95% CI)	
	Asthma incidence through first 4 y of life	Asthma incidence through first 11 y of life
**Mean PM_2.5_ over y 1-3**		
Individual characteristics		
Sex		
Female	1.34 (1.04-1.72)	1.22 (0.99-1.51)
Male	1.28 (0.98-1.66)	1.23 (0.99-1.53)
Education		
College and graduate school	1.24 (0.97-1.60)	1.17 (0.95-1.45)
High school	1.43 (1.07-1.90)	1.30 (1.02-1.65)
No high school	1.46 (0.96-2.20)	1.39 (0.97-1.99)
Race and ethnicity		
Black	1.60 (1.15-2.22)	1.41 (1.06-1.89)
Hispanic	1.37 (0.94-2.00)	1.20 (0.88-1.63)
White	1.17 (0.90-1.52)	1.15 (0.93-1.43)
Other^b^	1.48 (0.91-2.39)	1.51 (1.00-2.27)
Neighborhood characteristics		
% Black population		
Low	1.26 (0.97-1.63)	1.20 (0.97-1.50)
High	1.49 (1.02-2.18)	1.31 (0.93-1.84)
Population density		
Low	1.29 (0.99-1.66)	1.16 (0.93-1.44)
High	1.50 (0.81-2.78)	1.71 (1.03-2.84)
COI education		
Low	1.42 (1.08-1.88)	1.32 (1.04-1.67)
High	1.09 (0.81-1.47)	1.07 (0.83-1.37)
COI health environment		
Low	1.51 (1.15-1.98)	1.36 (1.08-1.71)
High	1.05 (0.79-1.41)	1.05 (0.82-1.34)
**Mean NO_2_ over y 1-3**		
Individual characteristics		
Sex		
Female	1.24 (1.00-1.53)	1.16 (0.97-1.38)
Male	1.27 (1.02-1.58)	1.30 (1.08-1.56)
Education		
College and graduate school	1.23 (1.00-1.52)	1.20 (1.01-1.43)
High school	1.26 (0.96-1.65)	1.23 (0.97-1.54)
No high school	1.32 (0.99-1.76)	1.32 (1.03-1.70)
Race and ethnicity		
Black	1.23 (0.95-1.59)	1.30 (1.03-1.63)
Hispanic	1.19 (0.87-1.63)	1.31 (1.00-1.72)
White	1.29 (0.97-1.71)	1.13 (0.90-1.41)
Other^b^	1.38 (0.81-2.33)	1.38 (0.86-2.20)
Neighborhood characteristics		
% Black population		
Low	1.17 (0.93-1.46)	1.15 (0.95-1.37)
High	1.43 (1.08-1.89)	1.41 (1.11-1.81)
Population density		
Low	1.24 (1.00-1.53)	1.16 (0.98-1.39)
High	1.31 (0.92-1.85)	1.46 (1.09-1.97)
COI education		
Low	1.16 (0.91-1.48)	1.25 (1.01-1.55)
High	1.35 (1.01-1.80)	1.16 (0.92-1.47)
COI health environment		
Low	1.23 (0.96-1.57)	1.23 (1.00-1.52)
High	1.28 (0.95-1.72)	1.22 (0.96-1.54)

Abbreviations: COI, Child Opportunity Index; HR, hazard ratio; NO_2_, nitrogen dioxide air pollution; PM_2.5_, fine particulate matter air pollution.

^a^ Results are presented as HRs and 95% CIs of asthma incidence through the first 4 and 11 years of life for a 1 IQR increase in each pollutant for individual-level and area-level modifiers.

^b^ The other race and ethnicity category included any race or ethnicity not categorized as Hispanic, non-Hispanic Black, or non-Hispanic White.

When we examined effect modification of air pollution averaged over the first 3 years of life by neighborhood factors (Table 3; eFigures 6 and 7 in Supplement 1), we found that for an IQR increase in PM_2.5_, children who resided in areas with lower education and health and environment opportunity had higher asthma incidence in the first 4 and 11 years of life. For an IQR increase in NO_2_, children who resided in areas with higher proportion of Black population and more urban areas had higher asthma incidence through the first 4 and 11 years of life. (Table 3)

In sensitivity analyses, the results were similar when we restricted analyses to those with documented wheeze symptoms during middle childhood following an asthma diagnosis by age 4 years (eFigure 8 in Supplement 1). In mixed-effect models with a random intercept for census tract in addition to cohort, the results did not change (eFigure 9 in Supplement 1). The multinomial regression results (eFigure 10 in Supplement 1) showed similar associations between PM_2.5_ and NO_2_ and asthma by age 4 years. The associations were weaker with asthma between ages 5 and 11, mostly for PM_2.5_.

## Discussion

In this multicohort study, we found that exposure to PM_2.5_ and NO_2_ during the first 3 years of life were associated with increased asthma incidence by early (<5 years) and middle (<12 years) childhood. Individual-level characteristics, including Black race and lower maternal educational attainment, and community-level factors, including lower health and environment child opportunity indices, population density, and neighborhoods with higher proportion of Black population, were associated with increased magnitude of the association between air pollution exposure and risk of childhood asthma.

Early childhood is a period of heightened concern, as higher exposures may lead to altered trajectories of airway and immune system development, with decreased lung function and asthma pathogenesis.^18,19^ The Tucson Children’s Respiratory Study found, with suggestion of a larger response in Black children, that higher childhood NO_2_ was associated with lower CC16, a biomarker in which its decrease has been associated with oxidative stress and reduced lung function. PM_2.5_ and NO_2_ may influence asthma development not only through oxidative stress leading to inflammation (eg, IL-6), but also through altered immune development, increased IgE-mediated allergic sensitization, and Th17-associated responses.^20,21^

By examining multiple periods of exposure and health outcomes,^5^ this analysis adds to the growing epidemiologic evidence that early-life air pollution exposures are associated with the onset of childhood asthma. In the Southern California Children’s Health Study (CHS)^22^ traffic-related pollution exposures during childhood, including NO_2_ at school^23^ and homes,^22^ were associated with increased asthma incidence. A follow-up study using 3 waves of the CHS Study^24^ found that decreases in NO_2_ and PM_2.5_ were significantly associated with lower asthma incidence. In Boston,^25^ first year of life and lifetime exposure to PM_2.5_ were associated with increased risk of pediatric asthma during early childhood (3-5 years of age). A previous analysis in the Cincinnati Childhood Allergy and Air Pollution Study (CCAAPS),^26^ found that exposure during the first 2 years of life, but not exposure during later childhood, was associated with asthma development by age 7 years. Another birth cohort study^27^ found increased risk of having an asthma diagnosis at age 13 years with NO_2_ postnatal exposure in the first year of life. Our study is consistent with these prior studies and suggests that the first 1 to 3 years of life are the most susceptible period for air pollution exposure to promote asthma development.

Our previous analysis^9^ of asthma incidence in the CREW consortium found that adverse neighborhood characteristics and Black race were associated with increased childhood asthma incidence. We interpreted race to signify measured and unmeasured exposures resulting from racism. Here, we leveraged the geographic, racial, and socioeconomic diversity of participant families and their communities to examine factors modifying associations between air pollution and asthma. We found that associations of air pollution exposure with asthma were elevated among Black children and children born to mothers without a high school diploma. These results are consistent with prior studies showing that socioeconomic position (SEP) and race are key drivers of elevated environmental exposures, including air pollution, and race and SEP, each of which can be independently or synergistically associated with elevated physiologic stress leading to inflammation that increases susceptibility to disease, including asthma.^28,29^ In addition, Black children are more likely to be exposed to adverse childhood experiences, poor housing quality and indoor environments, and have less access to healthy food and greenspace.^30^ These factors, likely in combination, may inequitably affect Black children such that the negative health effects due to exposure to air pollution are heightened.

Similar to individual-level race and SEP, we also found that neighborhood measures, including the percentage of Black population and lower health and environment COI, also were significant modifiers. The COI domain of health and environment represents the quality of neighborhoods in terms of housing, access to food, built environment, and exposure to pollution and heat.^15^ Previous studies have shown disparities in asthma prevalence and morbidity across communities related to violence, poor housing, elevated environmental exposures, lack of access to health care.^30^ The COI has been previously associated with population-level asthma morbidity in the US.^31^ A Swedish study using administrative data^32^ found that PM_2.5_ during the first 3  years of life increased asthma risk and that pollution-asthma associations were stronger in areas with lower education.

Our study has several strengths. To our knowledge, this is the first multicohort study of air pollution and asthma in the US that focused on the independent and interacting environmental and social influences on the age and child life-stage of asthma onset, demonstrating heightening of early-life pollution effects by adverse community-level exposures. We report findings that have a consistent pattern and are robust to different methods of statistically testing associations.^33^

The added value of this study includes cohorts that were specifically focused on measuring outcomes related to childhood asthma, with multiple decades of recruitment, varying in terms of the population selection criteria (general and high risk), and with socioeconomic, racial and ethnic, geographic and temporal diversity, and with a wide distribution of state-of-the-art modeling for spatio-temporally-resolved estimation of pollutants.

In Europe, a multicity study^34^ did not find significant association between air pollution exposure and childhood asthma. These cohorts were recruited in the late 1990s, and air pollution exposure was determined with land use regression models. In CREW, collecting and harmonizing data from each cohort enabled us to examine early and middle childhood onset of asthma as well as examine whether the associations differed by both individual-level and area-level factors in urban US settings. Given the longitudinal nature of the study, we were able to examine incident cases instead of a prevalence. The use of the DeGAUSS approach,^13,14^ a software containerization platform, enabled individual sites to geocode their birth addresses and then assign tract-level and exposure variables to those geocodes, so that all individual study sites had identically constructed data sets. Examining modification of air pollution on asthma by COI and SVI (indexes used in previous studies^35,36^ on body mass index and cardiometabolic risk) enabled us to characterize how composite, as well as individual metrics of community resources and risk were associated with worse pollution effects on childhood asthma.

### Limitations

This study had limitations. While the census variables are available every 10 years and have been merged to the nearest year of birth, SVI was available for the years 2000 and 2010 and COI only for 2010; therefore, some misclassification is possible. In these analyses, we examined NO_2_ and PM_2.5_, although other pollutants common in urban environments like ozone and other traffic-related air pollution may also contribute to asthma and interact with NO_2_ and PM_2.5_. Similarly, we do not have information about the contribution of pollution from indoor environments. While our analysis focused on early life exposures, future studies could test whether exposures in later life add cumulatively to lifetime risk of asthma.^25^

## Conclusions

In this cohort study of children from a highly diverse US population, PM_2.5_ and NO_2_ averaged over the first 3 years of life were associated with increased asthma incidence by early and middle childhood. The associations of PM_2.5_ and NO_2_ were greater among families living in urban US communities with fewer opportunities and resources with multiple environmental coexposures. Air pollution continues to be a global burden with serious consequences on childhood health. Reducing asthma risk in the US requires regulation and reduction of air pollution combined with creation of greater environmental, educational, and health equity at a community level.

## Notes

Supplement 1. **eMethods 1.** Description of the Participating CREW Cohorts **eMethods 2.** Supplementary Material and Methods **eTable 1.** Overview of Participating CREW Cohorts **eTable 2.** Child’s Demographic Characteristics and Caregiver Demographic Characteristics Among CREW Participants When Asthma Incidence Is Missing **eTable 3.** Child’s Demographic Characteristics, Caregiver Demographic Characteristics and Distribution of Respiratory Health Outcomes Among CREW Participants When PM_2.5 _Averaged Over Years 1-3 Is Missing **eTable 4.** Child’s Demographic Characteristics, Caregiver Demographic Characteristics and Distribution of Respiratory Health Outcomes Among CREW Participants When NO_2_ Averaged Over Years 1-3 Is Missing **eFigure 1.** Social Vulnerability Index (SVI) and Its Domains **eFigure 2.** Child Opportunity Index (COI) and Its Domains **eFigure 3.** Strobe Diagram of Analytical Cohort **eFigure 4.** Correlations Among PM_2.5_, NO_2_, U.S. Census Variables and COI and SVI **eFigure 5.** Odds Ratios (OR) of Asthma by Age 4 and 11 (A) and Hazard Ratios (HR) of Asthma Incidence (B) for PM_2.5_ and NO_2_ for the First Year of Life and Up to 4 Years and for the Averages of Years 1 and 2 and 1 Through 4 **eFigure 6.** Effect Modification of PM_2.5_ by Neighborhood Characteristics **eFigure 7.** Effect Modification of NO_2.5_ by Neighborhood Characteristics **eFigure 8.** Sensitivity Analysis: Odds Ratio of Asthma by Age 4 With Persistent Wheeze for One IQR Increase in Each Exposure Average **eFigure 9.** Odds Ratios (OR) of Asthma by Age 4 and Ages 5-11 for an IQR Increase in PM_2.5_ and NO_2_ for the First Year of Life and for the Averages of Year 1 and 2 and 1 Through 4 Using a Multinomial Regression
eReferences

Supplement 2.
Nonauthor Collaborators

Supplement 3.
Data Sharing Statement
