# Supplementary material for: Early-Life Exposure to Air Pollution and Childhood Asthma Cumulative Incidence in the ECHO CREW Consortium
Source: JAMA Netw Open. 2024 Feb 28;7(2):e240535. doi: 10.1001/jamanetworkopen.2024.0535 (PMC10902721; doi:10.1001/jamanetworkopen.2024.0535)
Supplement: Supplement 1. — eMethods 1. Description of the Participating CREW Cohorts eMethods 2. Supplementary Material and Methods eTable 1. Overview of Participating CREW Cohorts eTable 2. Child’s Demographic Characteristics and Caregiver Demographic Characteristics Among CREW Participants When Asthma Incidence Is Missing eTable 3. Child’s Demographic Characteristics, Caregiver Demographic Characteristics and Distribution of Respiratory Health Outcomes Among CREW Participants When PM2.5 Averaged Over Years 1-3 Is Missing eTable 4. Child’s Demographic Characteristics, Caregiver Demographic Characteristics and Distribution of Respiratory Health Outcomes Among CREW Participants When NO2 Averaged Over Years 1-3 Is Missing eFigure 1. Social Vulnerability Index (SVI) and Its Domains eFigure 2. Child Opportunity Index (COI) and Its Domains eFigure 3. Strobe Diagram of Analytical Cohort eFigure 4. Correlations Among PM2.5, NO2, U.S. Census Variables and COI and SVI eFigure 5. Odds Ratios (OR) of Asthma by Age 4 and 11 (A) and Hazard Ratios (HR) of Asthma Incidence (B) for PM2.5 and NO2 for the First Year of Life and Up to 4 Years and for the Averages of Years 1 and 2 and 1 Through 4 eFigure 6. Effect Modification of PM2.5 by Neighborhood Characteristics eFigure 7. Effect Modification of NO2.5 by Neighborhood Characteristics eFigure 8. Sensitivity Analysis: Odds Ratio of Asthma by Age 4 With Persistent Wheeze for One IQR Increase in Each Exposure Average eFigure 9. Odds Ratios (OR) of Asthma by Age 4 and Ages 5-11 for an IQR Increase in PM2.5 and NO2 for the First Year of Life and for the Averages of Year 1 and 2 and 1 Through 4 Using a Multinomial Regression eReferences [file jamanetwopen-e240535-s001.pdf]

Supplemental Online Content

Zanobetti A, Ryan PH, Coull BA, et al; ECHO Children's Respiratory and Environmental Workgroup. Early-life exposure to air pollution and childhood asthma cumulative incidence in the ECHO/CREW consortium. *JAMA Netw Open*. 2024;7(2):e240535. doi:10.1001/jamanetworkopen.2024.0535

eMethods 1. Description of the Participating CREW Cohorts

eMethods 2. Supplementary Material and Methods

eTable 1. Overview of Participating CREW Cohorts.

eTable 2. Child's Demographic Characteristics and Caregiver Demographic Characteristics Among CREW Participants When Asthma Incidence Is Missing

eTable 3. Child's Demographic Characteristics, Caregiver Demographic Characteristics and Distribution of Respiratory Health Outcomes Among CREW Participants When $PM_{2.5}$ Averaged Over Years 1-3 Is Missing

eTable 4. Child's Demographic Characteristics, Caregiver Demographic Characteristics and Distribution of Respiratory Health Outcomes Among CREW Participants When NO_2 Averaged Over Years 1-3 Is Missing

eFigure 1. Social Vulnerability Index (SVI) and Its Domains

eFigure 2. Child Opportunity Index (COI) and Its Domains

eFigure 3. Strobe Diagram of Analytical Cohort

eFigure 4. Correlations Among $PM_{2.5}$, NO_2, U.S. Census Variables and COI and SVI

eFigure 5. Odds Ratios (OR) of Asthma by Age 4 and 11 (A) and Hazard Ratios (HR) of Asthma Incidence (B) for $PM_{2.5}$ and NO_2 for the First Year of Life and Up to 4 Years and for the Averages of Years 1 and 2 and 1 Through 4

eFigure 6. Effect Modification of $PM_{2.5}$ by Neighborhood Characteristics

eFigure 7. Effect Modification of $NO_{2.5}$ by Neighborhood Characteristics

eFigure 8. Sensitivity Analysis: Odds Ratio of Asthma by Age 4 With Persistent Wheeze for One IQR Increase in Each Exposure Average

eFigure 9. Odds Ratios (OR) of Asthma by Age 4 and Ages 5-11 for an IQR Increase in $PM_{2.5}$ and NO_2 for the First Year of Life and for the Averages of Year 1 and 2 and 1 Through 4 Using a Multinomial Regression

eReferences

This supplemental material has been provided by the authors to give readers additional information about their work.

© 2024 Zanobetti A et al. *JAMA Network Open.*

eMethods 1: Description of the participating CREW cohorts

Our study population included 8 of the 12 longitudinal birth cohorts participating in CREW. Each cohort was originally established to identify prenatal and/or early life exposures that influence the development of asthma[1]. eTable 1 present an overview of the 8 participating CREW the cohorts, with the recruitment years, location and the population characteristics. The study population is diverse in terms of years of recruitment (1987–2007), and geographic locations of study participants, with representation from the East Coast (Baltimore, Boston, New York City), Midwest (Cincinnati, Detroit, Madison), and South (St. Louis). The cohorts were selected to represent a general population (CAS, CCCEH, IIS, WHEALS) or populations at higher risk for asthma (CCAAPS, COAST, EHAAS, URECA) on the basis of parental history of allergies or asthma. The cohort exclusion criteria for this CREW analysis included (1) not having pollution exposure estimates for the first 3 years of life (i.e., the oldest CREW cohort: TCRS, Tucson Children's Respiratory Study); and/or (2) not yet having harmonized asthma outcomes (i.e., the youngest CREW cohorts: MAAP, Microbes, Allergy, Asthma and Pets Study, INSPIRE, Infant Susceptibility to Pulmonary Infections and Asthma Following RSV Exposure study, WISC, Wisconsin Infant Study Cohort)

© 2024 Zanobetti A et al. *JAMA Network Open.*

eMethods 2: Supplementary Material and Methods

Exposure assessment: $PM_{2.5}$ and NO_2

We applied previously developed and validated prediction models[2–4] to estimate $PM_{2.5}$ and NO_2 exposures for CREW participants at their home addresses. Briefly, daily estimates of $PM_{2.5}$ and NO_2 from 2000-2016 were generated at a 1 km^2 resolution using a neural network model incorporating satellite-based measurements, simulation outputs from a chemical transport model, land-use terms, meteorology, and monitoring data[2,3]. We also obtained monthly $PM_{2.5}$ predictions at the 6 km^2 grid for the years 1988-2007 from a spatiotemporal generalized additive mixed model based on monitoring data, the Interagency Monitoring of Protected Visual Environments (IMPROVE) network, time-varying spatial smooths of monitoring site geographic coordinates, GIS-based time-invariant geographical covariates, and meteorological covariates[4].

The 1 km^2 estimates were linked to the home addresses for each CREW participant using our Decentralized Geomarker Assessment for Multi-site Studies (DeGAUSS) software[5,6] approach. Briefly, each CREW cohort compiled and geocoded the longitudinal address history for each participant, beginning prenatally through their most recently available address. Data managers at each cohort applied a DeGAUSS container to link geocoded addresses and dates to 1 km^2 estimates of $PM_{2.5}$ and NO_2 to generate daily estimates of exposure for each participant beginning with the move-in date and ending with the move-out date for each address. For the monthly $PM_{2.5}$ predictions at the 6 km^2 grid for the years 1988-2007 participants' geocodes were spatially joined with the grid centroids using a script written in the R 4.4.1 project for statistical computing software (https://www.r-project.org/), and $PM_{2.5}$ estimates were merged via SAS 9.4 analytical software (https://www.sas.com/).

© 2024 Zanobetti A et al. *JAMA Network Open.*

For cohorts with person-time before 2000, we averaged the daily 1 km^2 data over person months to match the monthly 6 km^2 metric and used linear regression models on monthly averages to create cohort-specific calibration factors, using all available data for the overlap timeframe 2000-2007.

Based on a crosswalk file between 1 km^2 and 6 km^2 centroids, the daily 1 km^2 data were spatially averaged over the 6 km^2 grid and temporally averaged over calendar month. We applied cohort-specific regression models of the form:

$$PM25\_month\_1km\_avg_{sm} = \alpha + \beta * PM25\_month\_6km_{sm} + \varepsilon_{sm} \qquad (1)$$

where s is the index for the subject and m for month. The coefficients α and β were estimated via Deming approach[7], a regression technique for two-dimensional data where both variables are measured with error. Compared to ordinary (least-squares) regression, where the sum of the squares of residual errors is minimized in the Y direction, in the Deming regression model, the sum of the squares of the perpendicular distances from the data points to the line is minimized.

Using the estimated regression coefficients from model (1), we calibrated the 6 km^2 annual, monthly, and prenatal exposures to the 1 km^2 level and used 1 km^2 exposure when available and the calibrated 6 km^2 exposure estimates for time periods before 2000.

Neighborhood-level characteristics

We obtained U.S. census data and boundary files for the years 1980, 1990, 2000, and 2010 for % population with low income, % Black population, population density (population per km^2), median household income (US Dollars [USD]) and % low-income families. We also obtained the 2010 COI and the SVI for the years 2000 and 2010.

© 2024 Zanobetti A et al. *JAMA Network Open.*

COI[8] is a measure that describes and quantifies the quality of neighborhoods in which U.S. children live. It combines public data from 29 neighborhood-level indicators into an overall COI score and grouped into 3 domains--education, health and environment, and social and economic--for almost all U.S. Census tracts (eFigure 1). The scores were standardized at the national level, with higher scores reflecting more favorable neighborhood opportunities relative to other neighborhoods across the U.S.

SVI[9] is a measure of socially vulnerable populations especially at risk during public health emergencies due to of factors like socioeconomic status, household characteristics, minoritized racial and ethnic status, or housing type and transportation. SVI is computed at the census tract level from 15 U.S. Census variables as percentile ranks, ranging from 0 (lowest vulnerability) to 1 (highest vulnerability) and available as an overall SVI combining the 15 variables, grouped into 4 domains: socioeconomic status, household composition and disability, minoritized racial and ethnic and language status, and housing and transportation type. (eFigure 2).

Using census tracts of each participant's address obtained with the DeGAUSS software[10], we merged U.S. Census data, COI and SVI to the nearest year of birth for each participant.

eTable 1. Overview of Participating CREW Cohorts.

Cohort	Recruitment years	Location	Population
CAS	1987-1989	Suburban Detroit	General
CCAAPS	2001-2003	Cincinnati	High risk
CCCEH	1998-2006	Manhattan and Bronx	General
COAST	1998-2000	Madison	High risk
EHAAS	1994-1996	Boston	High risk
IIS	1997-2003	Tucson	General
URECA			
Baltimore	2004-2006	Baltimore	High risk, urban
Boston	2004-2006	Boston	High risk, urban
New York	2004-2006	New York	High risk, urban
St Louis	2004-2006	St Louis	High risk
WHEALS	2003-2007	Metro Detroit	General

© 2024 Zanobetti A et al. *JAMA Network Open.*

eTable 2: Child's demographic characteristics and caregiver demographic characteristics among CREW participants when asthma incidence is missing

		CAS	CCAAPS	CCCEH	COAST	EHAAS	IIS	URECA Baltimore	URECA Boston	URECA New York	URECA St Louis	WHEALS	Total
Child sex													
Male	N	1	41	15	12		1	10	15	10	16	68	189
	%	50.0	58.6	55.6	54.5		50.0	50.0	51.7	47.6	45.7	47.6	50.9
Female	N	1	29	12	10		1	10	14	11	19	75	182
	%	50.0	41.4	44.4	45.5		50.0	50.0	48.3	52.4	54.3	52.4	49.1
Child race/ethnicity													
White	N	1	53	0	20		1	2	3	0	4	29	113
	%	50.0	75.7	0.0	90.9		50.0	10.0	10.3	0.0	11.4	20.3	30.5
Hispanic	N	0	0	22	0		0	2	8	12	1	2	47
	%	0.0	0.0	81.5	0.0		0.0	10.0	27.6	57.1	2.9	1.4	12.7
Black	N	1	11	5	1		0	16	16	9	28	107	194
	%	50.0	15.7	18.5	4.5		0.0	80.0	55.2	42.9	80.0	74.8	52.3
Others	N	0	6	0	1		0	0	2	0	2	5	16
	%	0.0	8.6	0.0	4.5		0.0	0.0	6.9	0.0	5.7	3.5	4.3
Mother's education	%												
College and graduate	N	1	50	6	20		1	2	5	3	5	99	192
	%	50.0	71.4	22.2	90.9		50.0	10.0	17.2	14.3	14.3	69.2	51.8
HS	N	1	15	11	2		0	0	5	2	11	32	79
	%	50.0	21.4	40.7	9.1		0.0	0.0	17.2	9.5	31.4	22.4	21.3
No HS	N	0	5	10	0		0	18	19	16	19	12	99
	%	0.0	7.1	37.0	0.0		0.0	90.0	65.5	76.2	54.3	8.4	26.7
Parental history of Asthma	N	1	23	8	13		1	13	19	15	21	39	153
	%	50.0	32.9	29.6	59.1		50.0	65.0	65.5	71.4	60.0	27.3	41.2
Smoking during pregnancy	N	0	11	0	0		1	5	3	1	5	22	48
	%	0.0	15.7	0.0	0.0		50.0	25.0	10.3	4.8	14.3	15.4	12.9
Total		2	70	27	22		2	20	29	21	35	143	371

© 2024 Zanobetti A et al. *JAMA Network Open.*

eTable 3: Child's demographic characteristics, caregiver demographic characteristics and distribution of respiratory health outcomes among CREW participants when PM2.5 averaged over years 1-3 is missing

		CAS	CCAAPS	CCCEH	COAST	EHAAS	IIS	URECA Baltimore	URECA Boston	URECA New York	URECA St Louis	WHEALS	Total
Child sex													
Male	N	91	6	158	6	2	1	0	0	1	3	130	398
	%	49.7	66.7	53.6	100.0	28.6	100.0	0.0	0.0	33.3	42.9	47.4	50.6
Female	N	92	3	137	0	5	0	1	1	2	4	144	389
	%	50.3	33.3	46.4	0.0	71.4	0.0	100.0	100.0	66.7	57.1	52.6	49.4
Child race/ethnicity													
White	N	168	5	0	5	1	1	0	0	0	0	68	248
	%	91.8	55.6	0.0	83.3	14.3	100.0	0.0	0.0	0.0	0.0	24.8	31.5
Hispanic	N	4	1	198	0	4	0	0	0	3	0	9	219
	%	2.2	11.1	67.1	0.0	57.1	0.0	0.0	0.0	100.0	0.0	3.3	27.8
Black	N	2	2	97	1	1	0	1	1	0	6	185	296
	%	1.1	22.2	32.9	16.7	14.3	0.0	100.0	100.0	0.0	85.7	67.5	37.6
Others	N	9	1	0	0	1	0	0	0	0	1	12	24
	%	4.9	11.1	0.0	0.0	14.3	0.0	0.0	0.0	0.0	14.3	4.4	3.0
Mother's education	%												
College and graduate	N	113	6	76	5	3	1	0	1	1	1	192	399
	%	61.7	66.7	25.8	83.3	42.9	100.0	0.0	100.0	33.3	14.3	70.1	50.7
HS	N	60	3	116	0	3	0	0	0	2	0	56	240
	%	32.8	33.3	39.3	0.0	42.9	0.0	0.0	0.0	66.7	0.0	20.4	30.5
No HS	N	10	0	103	1	1	0	1	0	0	6	26	148
	%	5.5	0.0	34.9	16.7	14.3	0.0	100.0	0.0	0.0	85.7	9.5	18.8
Parental history of Asthma	N	23	4	100	5	6	0	0	1	2	5	84	230
	%	12.6	44.4	33.9	83.3	85.7	0.0	0.0	100.0	66.7	71.4	30.7	29.2
Smoking during pregnancy	N	27	2	7	1	0	0	0	0	0	0	37	74
	%	14.8	22.2	2.4	16.7	0.0	0.0	0.0	0.0	0.0	0.0	13.5	9.4
Asthma by age 11	N	35	1	104	1	1	0	0	1	1	6	23	173
	%	19.1	11.1	35.3	16.7	14.3	0.0	0.0	100.0	33.3	85.7	8.4	22.0
Asthma by age 4	N	19	1	77	1	1	0	0	1	1	6	21	128
	%	10.4	11.1	26.1	16.7	14.3	0.0	0.0	100.0	33.3	85.7	7.7	16.3
Total		183	9	295	6	7	1	1	1	3	7	274	787

© 2024 Zanobetti A et al. *JAMA Network Open.*

eTable 4: Child's demographic characteristics, caregiver demographic characteristics and distribution of respiratory health outcomes among CREW participants when NO2 averaged over years 1-3 is missing

		CAS	CCAAPS	CCCEH	COAST	EHAAS	IIS	URECA Baltimore	URECA Boston	URECA New York	URECA St Louis	WHEALS	Total
Child sex													
Male	N	365	6	187	82	260	79	0	0	1	3	130	1113
	%	48.7	66.7	52.1	55.8	53.4	47.0	0.0	0.0	33.3	42.9	47.4	50.5
Female	N	385	3	172	65	227	89	1	1	2	4	144	1093
	%	51.3	33.3	47.9	44.2	46.6	53.0	100.0	100.0	66.7	57.1	52.6	49.5
Child race/ethnicity													
White	N	685	5	0	135	387	101	0	0	0	0	68	1381
	%	91.3	55.6	0.0	91.8	79.5	60.1	0.0	0.0	0.0	0.0	24.8	62.6
Hispanic	N	20	1	225	3	37	52	0	0	3	0	9	350
	%	2.7	11.1	62.7	2.0	7.6	31.0	0.0	0.0	100.0	0.0	3.3	15.9
Black	N	11	2	134	7	33	5	1	1	0	6	185	385
	%	1.5	22.2	37.3	4.8	6.8	3.0	100.0	100.0	0.0	85.7	67.5	17.5
Others	N	34	1	0	2	30	10	0	0	0	1	12	90
	%	4.5	11.1	0.0	1.4	6.2	6.0	0.0	0.0	0.0	14.3	4.4	4.1
Mother's education	%												
College and graduate	N	469	6	84	134	452	121	0	1	1	1	192	1461
	%	62.5	66.7	23.4	91.2	92.8	72.0	0.0	100.0	33.3	14.3	70.1	66.2
HS	N	257	3	150	9	29	33	0	0	2	0	56	539
	%	34.3	33.3	41.8	6.1	6.0	19.6	0.0	0.0	66.7	0.0	20.4	24.4
No HS	N	24	0	125	4	6	14	1	0	0	6	26	206
	%	3.2	0.0	34.8	2.7	1.2	8.3	100.0	0.0	0.0	85.7	9.5	9.3
Parental history of Asthma	N	105	4	120	99	250	54	0	1	2	5	84	724
	%	14.0	44.4	33.4	67.3	51.3	32.1	0.0	100.0	66.7	71.4	30.7	32.8
Smoking during pregnancy	N	123	2	8	8	30	22	0	0	0	0	37	230
	%	16.4	22.2	2.2	5.4	6.2	13.1	0.0	0.0	0.0	0.0	13.5	10.4
Asthma by age 11	N	122	1	135	68	163	25	0	1	1	6	23	545
	%	16.3	11.1	37.6	46.3	33.5	14.9	0.0	100.0	33.3	85.7	8.4	24.7
Asthma by age 4	N	68	1	98	27	125	20	0	1	1	6	21	368
	%	9.1	11.1	27.3	18.4	25.7	11.9	0.0	100.0	33.3	85.7	7.7	16.7
Total		750	9	359	147	487	168	1	1	3	7	274	2206

© 2024 Zanobetti A et al. *JAMA Network Open.*

eFigure 1: Social Vulnerability Index (SVI) and its domains.

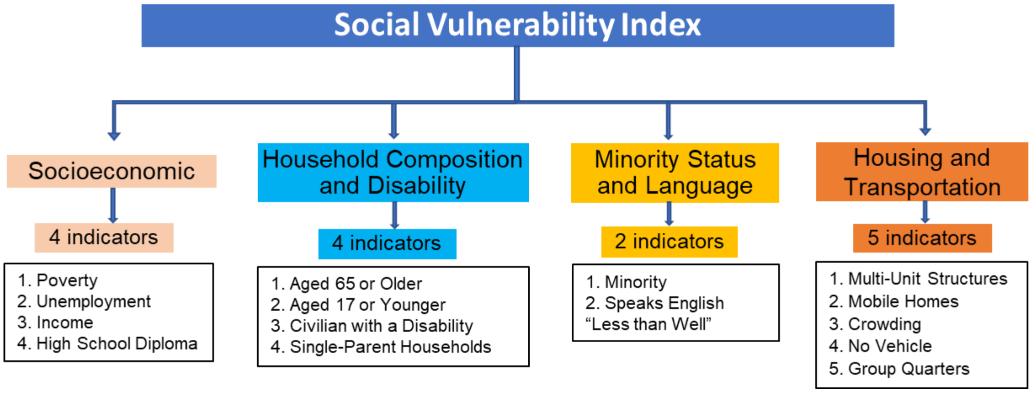

© 2024 Zanobetti A et al. *JAMA Network Open.*

eFigure 2: Child Opportunity Index (COI) and its domains.

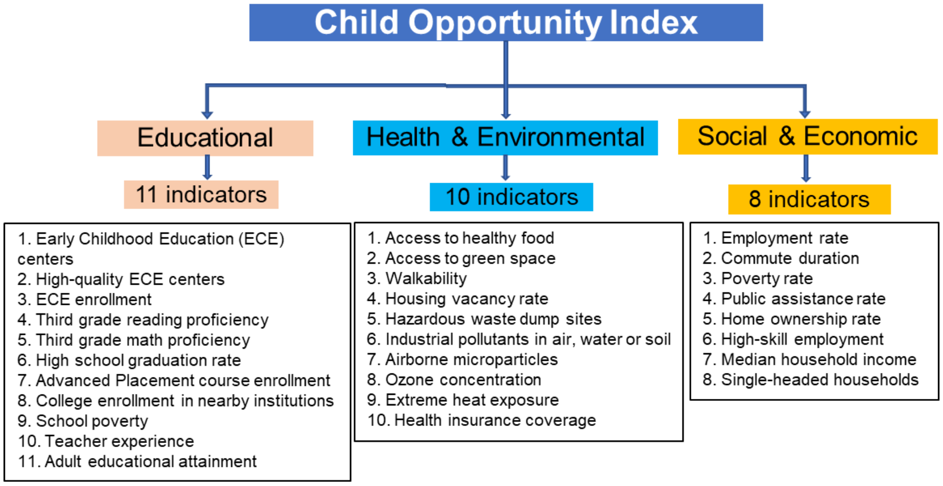

© 2024 Zanobetti A et al. *JAMA Network Open.*

eFigure 3: Strobe diagram of analytical cohort

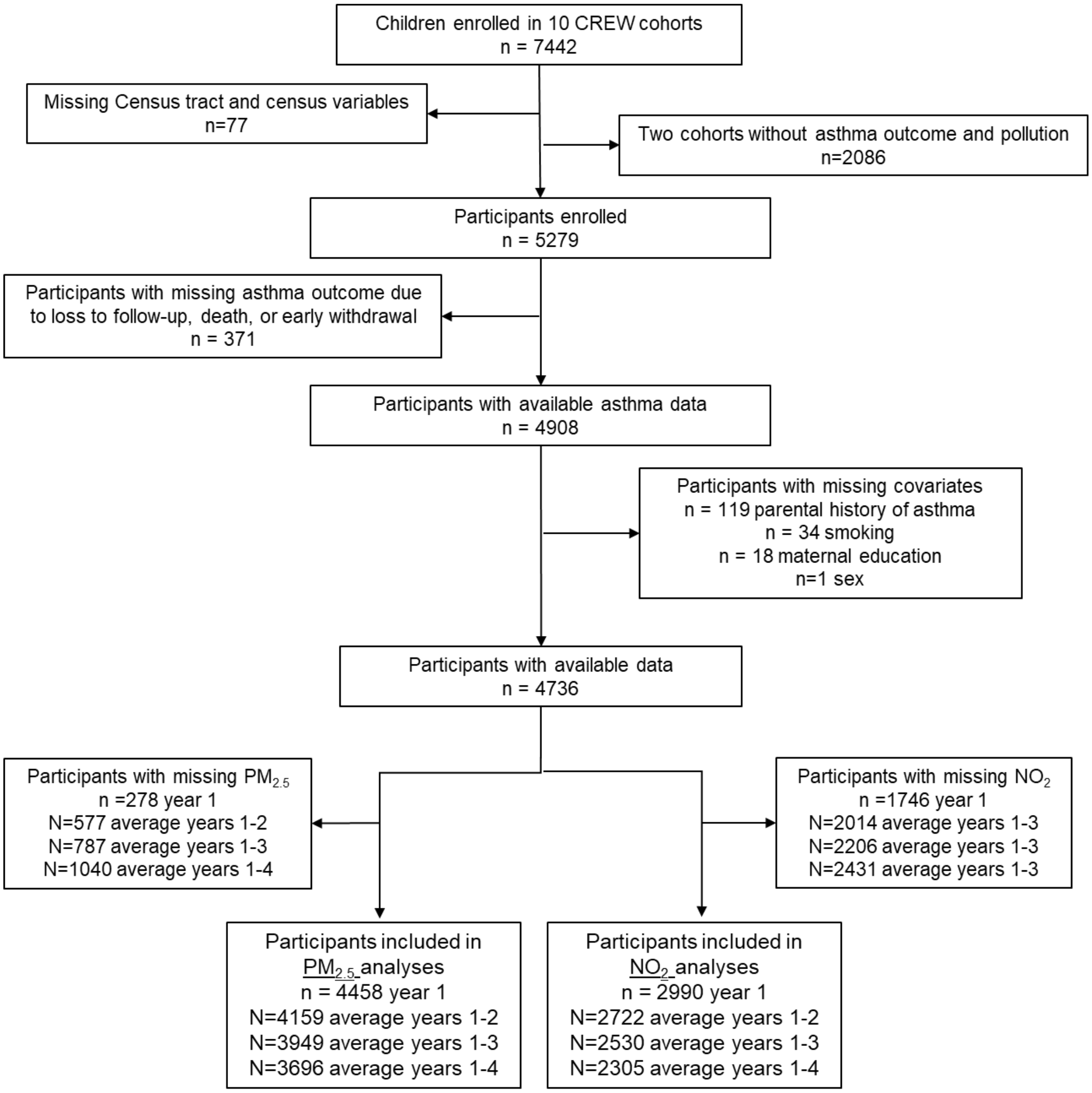

© 2024 Zanobetti A et al. *JAMA Network Open.*

eFigure 4: Correlations among $PM_{2.5}$, NO_2, U.S. Census variables and COI and SVI*.

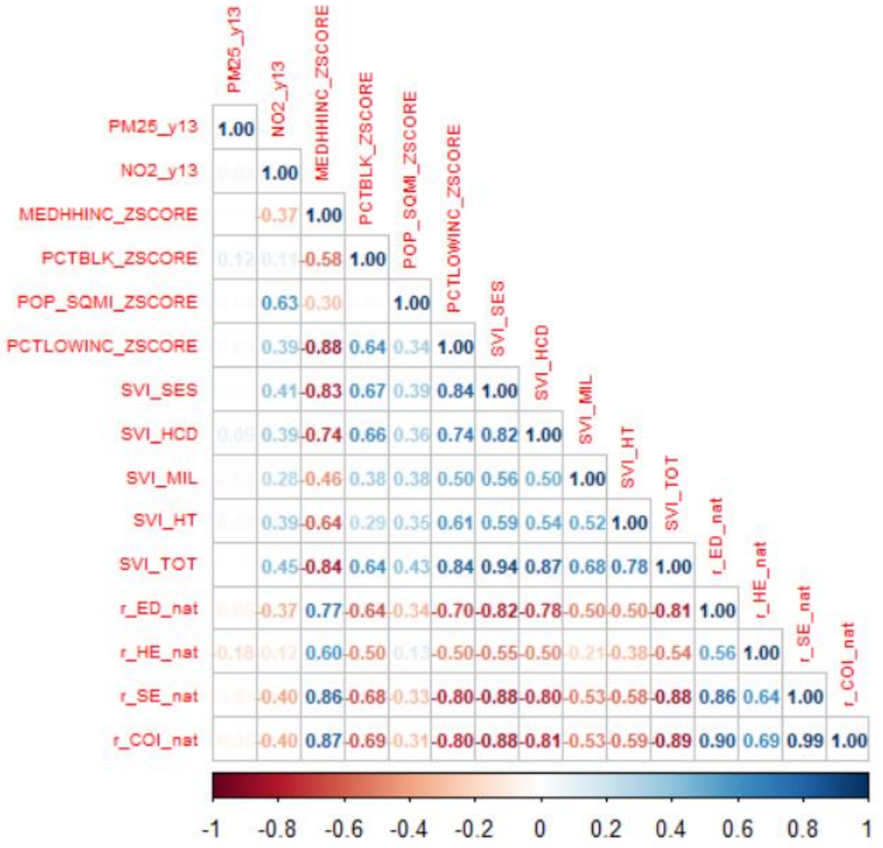

*Abbreviations: PM25_13: PM2.5 averaged over years 1-3; NO2_13: NO2 averaged over years 1-3; MEDHHINC_ZSCORE: Median household income z-score; PCTBLK_ZSCORE: Percent Black Population z-score; POP_SQMI_ZSCORE: Population per square mile z-score; PCTLOWINC_ZSCORE: Percent Low income population z-score; SVI_SES: SVI Socioeconomic domain; SVI_HCD: SVI Household Composition and Disability domain; SVI_MIL: SVI minority and Language status domain; SVI_HT: SVI Housing and Transportation domain; SVI_TOT: overall SVI; r_ED_nat: Nationally-normed COI for the Education domain. r_HE_nat Nationally-normed COI for the Health and Environment domain, r_SE_nat Nationally-normed COI for the Social and Economic domain., r_COI_nat Nationally-normed COI for the overall COI.

© 2024 Zanobetti A et al. *JAMA Network Open.*

eFigure 5: Odds Ratios (OR) of asthma by age 4 and 11 (A) and Hazard Ratios (HR) of asthma incidence (B) for $PM_{2.5}$ and NO_2 for prenatal, the first year of life and up to 4 years and for the averages of years 1 and 2 and 1 through 4.

A

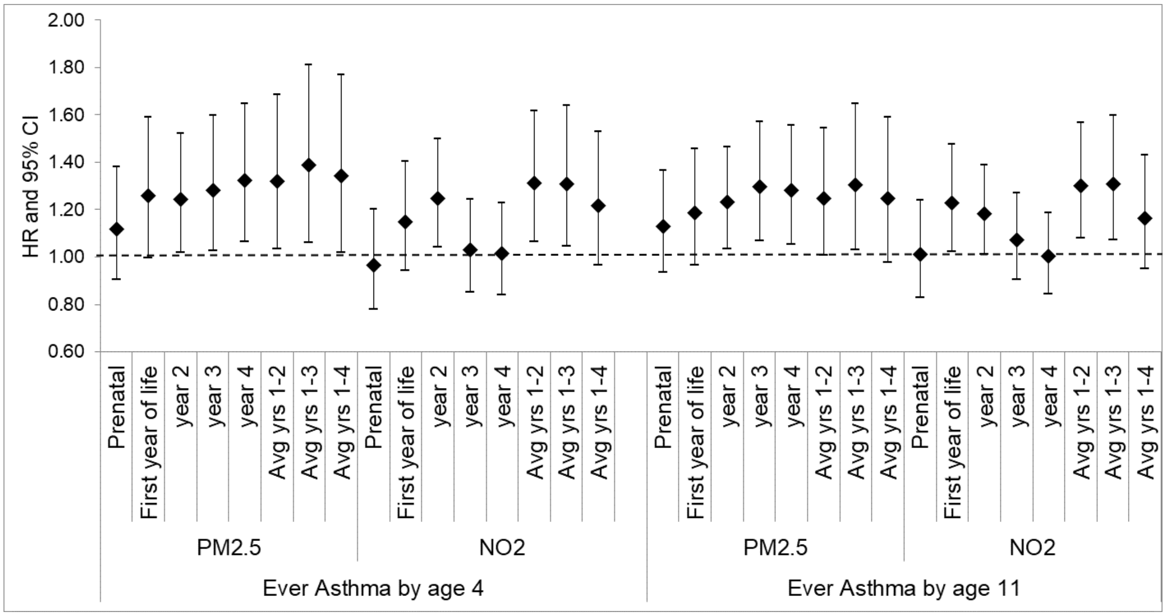

B

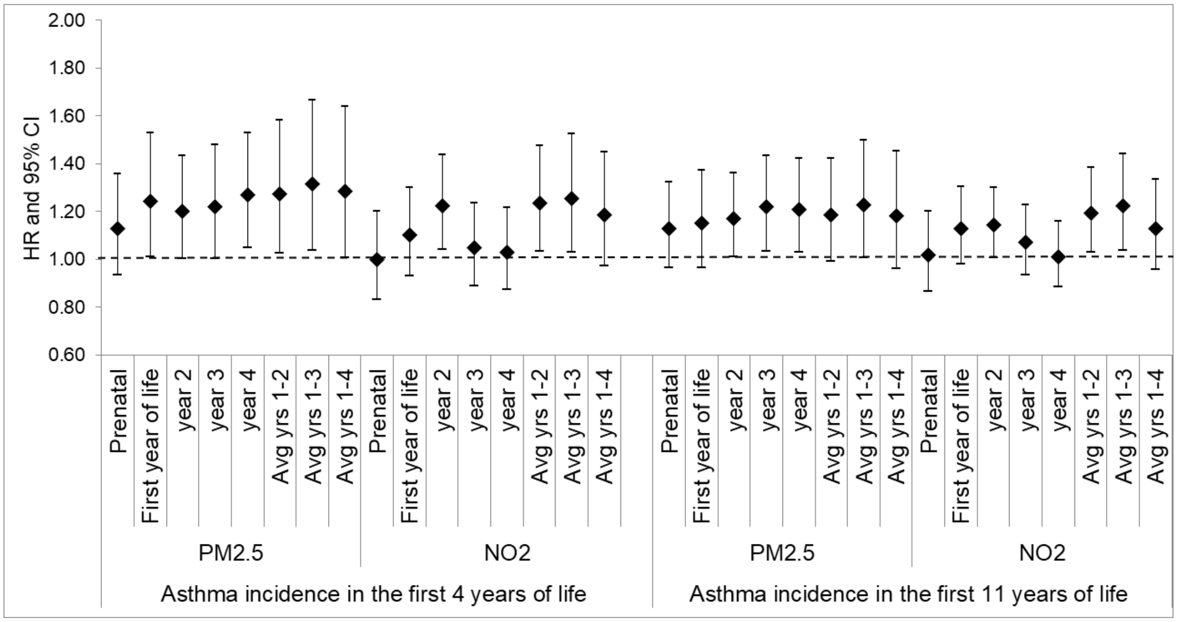

© 2024 Zanobetti A et al. *JAMA Network Open.*

eFigure 6: Effect modification of $PM_{2.5}$ averaged over years 1-3 by neighborhood characteristics. Results are presented as Hazard Ratios and 95% CI of asthma incidence in the first 4 (A) and 11 (B) years of life for an IQR increase in $PM_{2.5}$ computed at the 10th (Low) and 90th (High) percentile of each neighborhood characteristics.

A

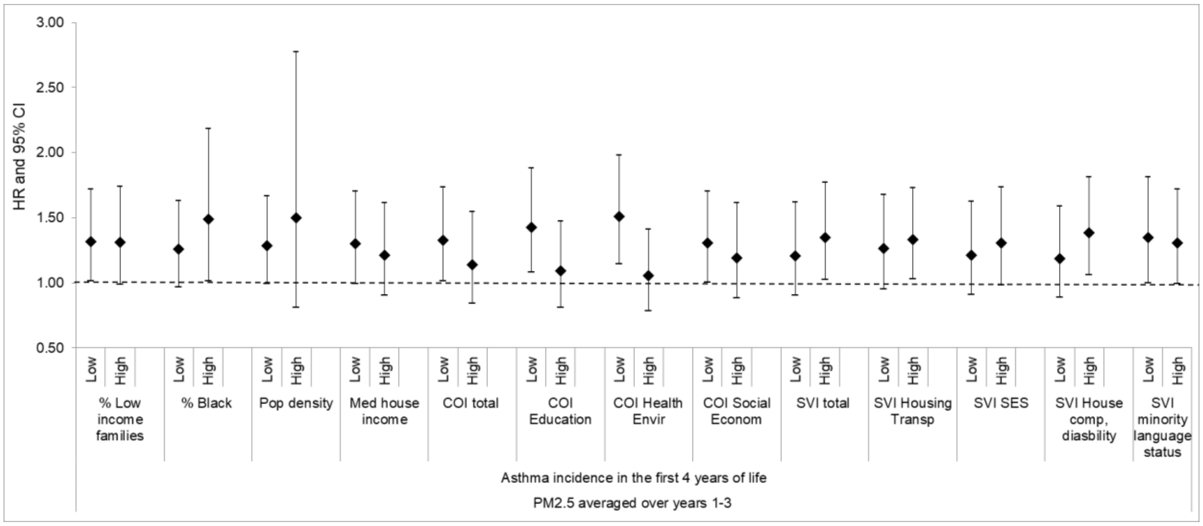

B

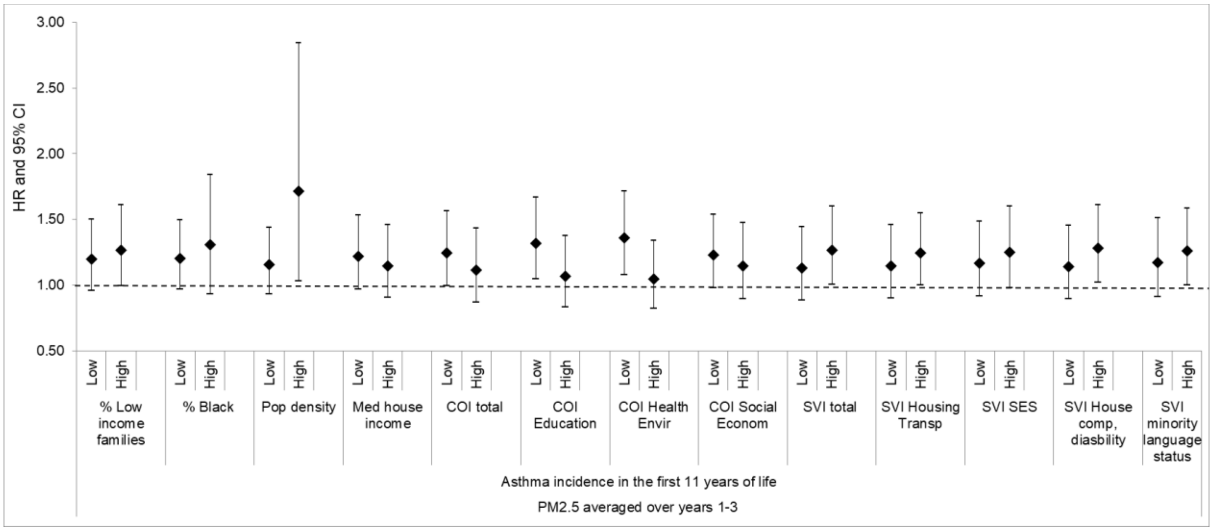

© 2024 Zanobetti A et al. *JAMA Network Open.*

eFigure 7: Effect modification of NO_2 averaged over years 1-3 by neighborhood characteristics. Results are presented as Hazard Ratios and 95% CI of asthma incidence in the first 4 (A) and 11 (B) years of life for an IQR increase in NO_2 computed at the 10th (Low) and 90th (High) percentile of each neighborhood characteristics.

A

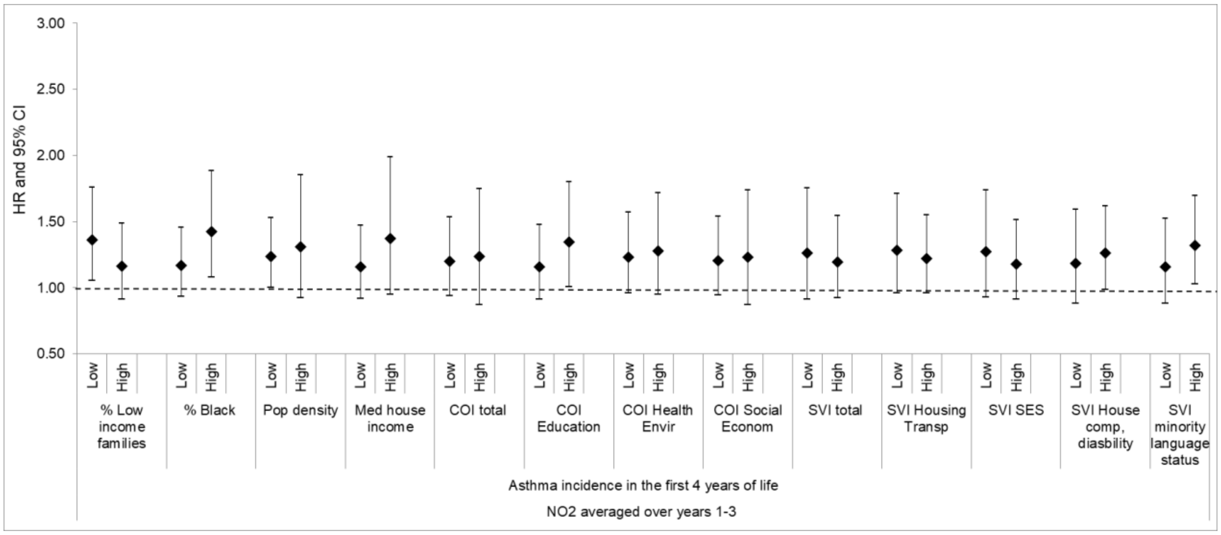

B

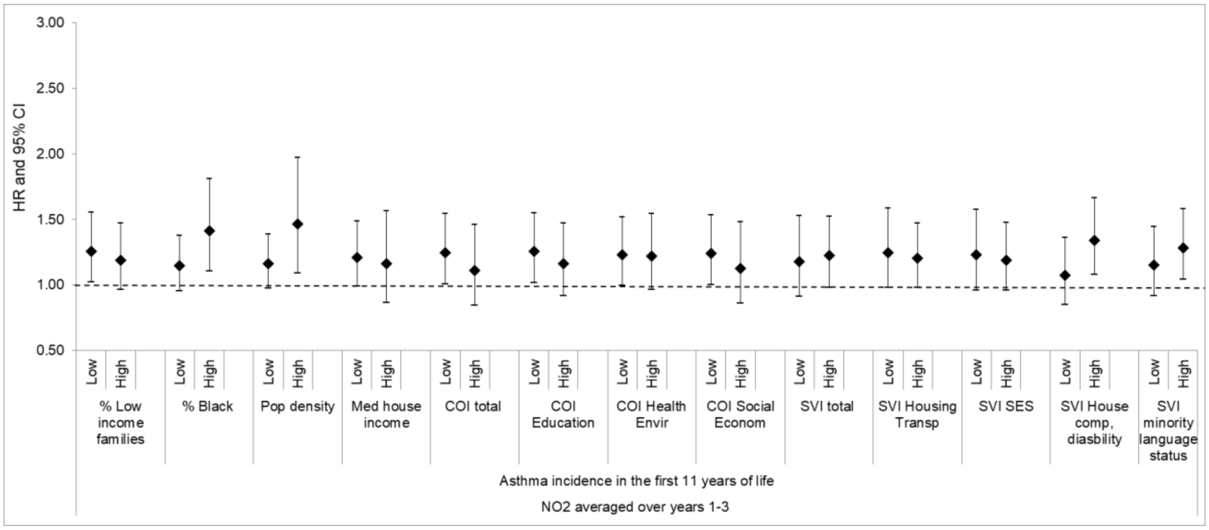

© 2024 Zanobetti A et al. *JAMA Network Open.*

eFigure 8. Sensitivity analysis: Hazard Ratio of asthma incidence in the first 4 years of life with persistent wheeze for one IQR increase in each exposure average

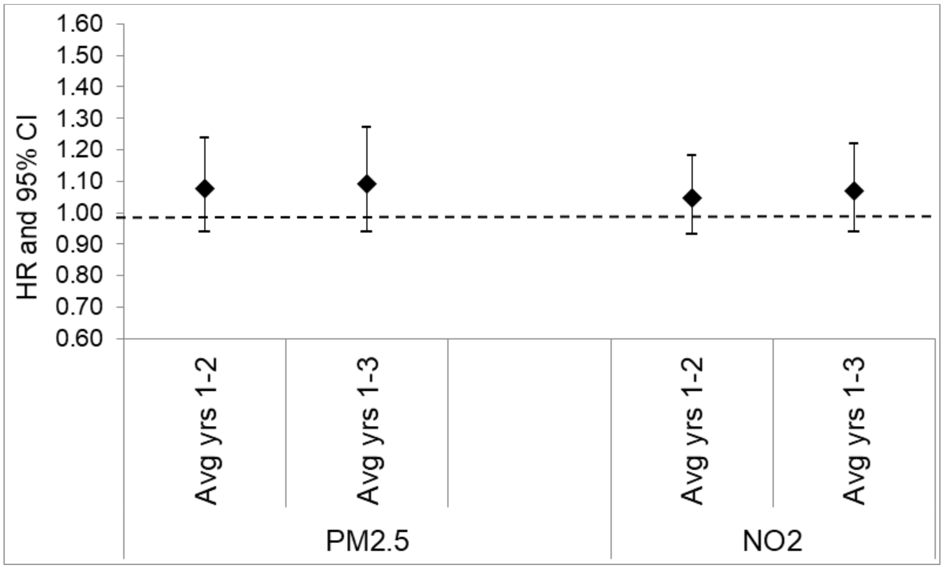

© 2024 Zanobetti A et al. *JAMA Network Open.*

eFigure 9: Sensitivity analysis results using mixed effect models with adjustment for census tract as a random intercept in addition to cohort. Hazard Ratios (HR) of asthma incidence (A) and Odds Ratios (OR) of ever asthma by age 4 and 11 (B) for $PM_{2.5}$ and NO_2 for prenatal, first year of life and up to 4 previous years and for the averages of years 1-2 and up to 1 through 4.

A

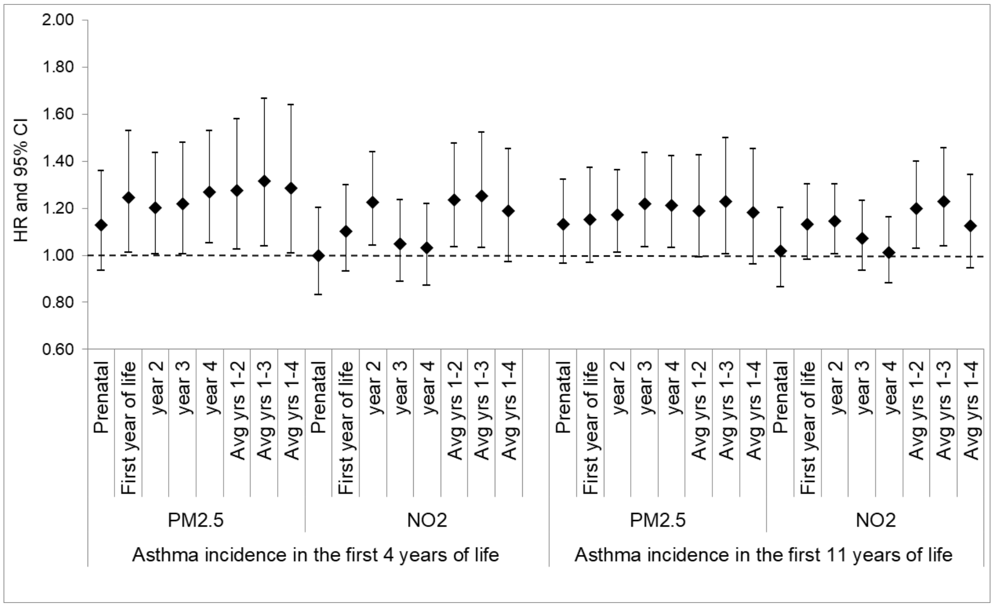

B

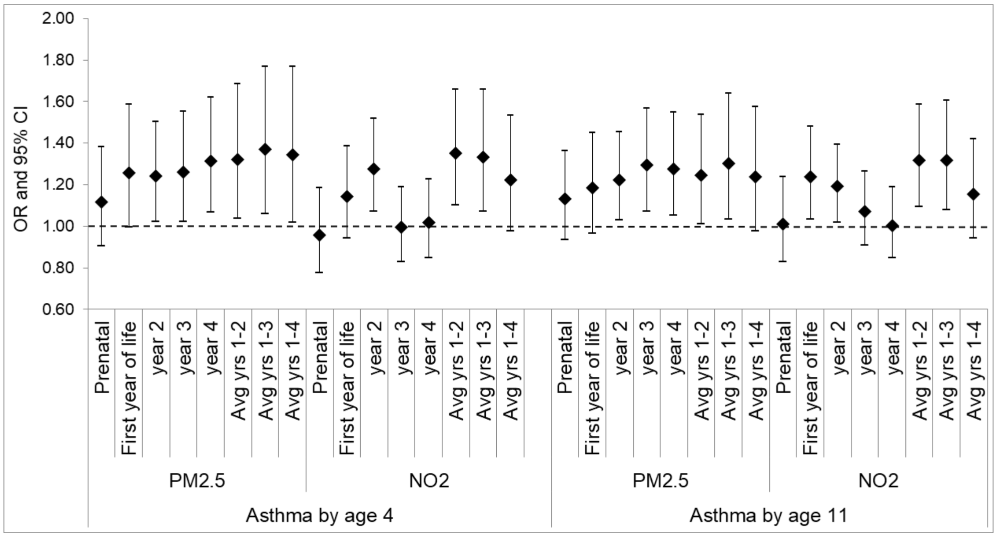

© 2024 Zanobetti A et al. *JAMA Network Open.*

Figure 10: Odds Ratios (OR) of asthma by age 4 and ages 5-11 for an IQR increase in $PM_{2.5}$ and NO_2 for the first year of life and for the averages of year 1 and 2 and 1 through 4 using a multinomial regression.

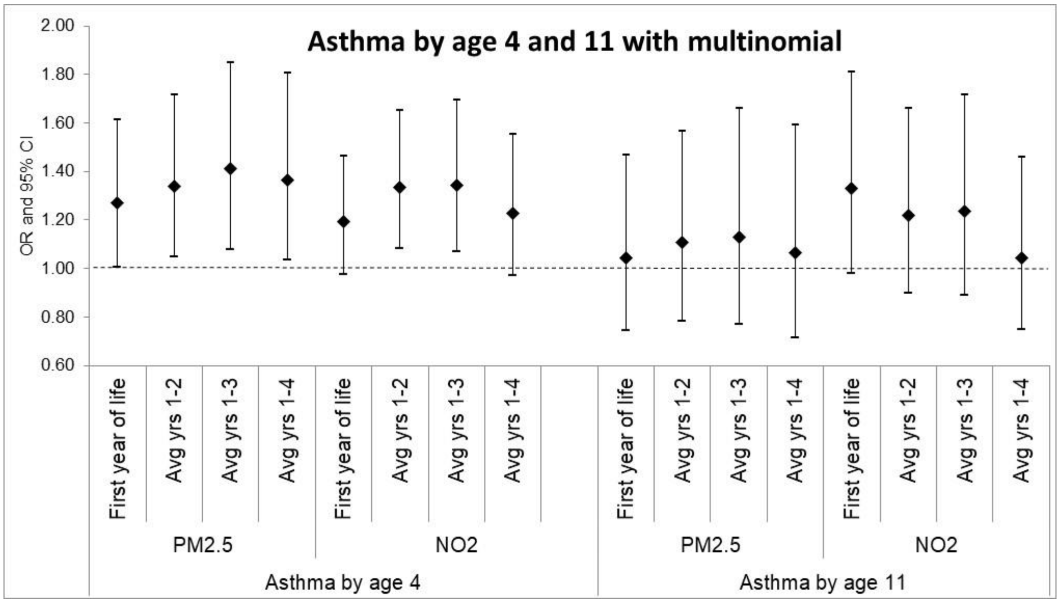

© 2024 Zanobetti A et al. *JAMA Network Open.*

eReferences

1. Gern JE, Jackson DJ, Lemanske RF, Seroogy CM, Tachinardi U, Craven M, Hwang SY, Hamilton CM, Huggins W, O'Connor GT, Gold DR, Miller R, Kattan M, Johnson CC, Ownby D, et al. The Children's Respiratory and Environmental Workgroup (CREW) birth cohort consortium: Design, methods, and study population. *Respiratory Research*. 2019;20(1).
2. Di Q, Rowland S, Koutrakis P, Schwartz J. A hybrid model for spatially and temporally resolved ozone exposures in the continental United States. *Journal of the Air & Waste Management Association*. 2017.
3. Di Q, Kloog I, Koutrakis P, Lyapustin A, Wang Y, Schwartz J. Assessing PM2.5 Exposures with High Spatiotemporal Resolution across the Continental United States. *Environmental Science and Technology*. 2016.
4. Yanosky JD, Paciorek CJ, Laden F, Hart JE, Puett RC, Liao D, Suh HH. Spatio-temporal modeling of particulate air pollution in the conterminous United States using geographic and meteorological predictors. *Environmental Health*. 2014;13:63.
5. Brokamp C. DeGAUSS: Decentralized Geomarker Assessment for Multi-Site Studies. *Journal of Open Source Software*. 2018;3(30):812.
6. Brokamp C, Wolfe C, Lingren T, Harley J, Ryan P. Decentralized and reproducible geocoding and characterization of community and environmental exposures for multisite studies. *Journal of the American Medical Informatics Association*. 2018;25(3):309–314.
7. Cornbleet PJ, Gochman N. Incorrect least-squares regression coefficients in method-comparison analysis. *Clinical Chemistry*. 1979;25(3):432–438.
8. Acevedo-Garcia D, Noelke C, McArdle N, Sofer N, Hardy EF, Weiner M, Baek M, Huntington N, Huber R, Reece J. Racial and ethnic inequities in children's neighborhoods: Evidence from the new child opportunity index 2.0. *Health Affairs*. 2020;39(10):1693–1701.
9. Flanagan BE, Hallisey EJ, Adams E, Lavery A. Measuring community vulnerability to natural and anthropogenic hazards: The Centers for Disease Control and Prevention's social vulnerability index. *Journal of Environmental Health*. 2018;80(10):34–36.
10. Ryan PH, Brokamp C, Blossom J, Lothrop N, Miller RL, Beamer PI, Visness CM, Zanobetti A, Andrews H, Bacharier LB, Hartert T, Johnson CC, Ownby D, Lemanske RF, Gibson H, et al. A Distributed Geospatial Approach to Describe Community Characteristics for Multi-Site Studies. *Journal of Clinical and Translational Science*. 2021.

© 2024 Zanobetti A et al. *JAMA Network Open.*
