# Supplementary material for: Early-Life Exposure to Air Pollution and Childhood Asthma Cumulative Incidence in the ECHO CREW Consortium
Source: JAMA Netw Open. 2024 Feb 28;7(2):e240535. doi: 10.1001/jamanetworkopen.2024.0535 (PMC10902721; doi:10.1001/jamanetworkopen.2024.0535)
Supplement: Supplement 2. — Nonauthor Collaborators [file jamanetwopen-e240535-s002.pdf]

Supplemental Online Content: Nonauthor Collaborators
*First name, last name, and suffix (if applicable) are required and will appear in PubMed.

***Group Name(s): ECHO Children's Respiratory and Environmental Workgroup (CREW) consortium**							
***First Name and Middle Initial(s)**	***Last Name**	***Suffix (eg, Jr, III)**	Academic Degrees	Institution	Location (city, state/province, country)	Role or Contribution, eg, chair, principal investigator	Group (if more than 1 Group listed in the byline) and/or Subgroup (eg, Steering Committee)
Nonna	Akkerman			Henry Ford Health	Detroit, MI		CAS
Elizabeth	Anderson			University of Wisconsin-Madison	Madison, WI		COAST
Mary Ann	Aubuchon			Henry Ford Health	Detroit, MI		WHEALS
Charles	Barone			Henry Ford Healths	Detroit, MI	Coinvestigator/Primary	WHEALS
Pam	Bates			Washington University School of Me	St Louis, MO	Study Coordinator	URECA
Jessica	Baucom			Rho, Inc.	Durham, NC	Project Manager	URECA
Patrice	Becker			National Institute of Allergy and Infe	Bethesda, MD		URECA
Stacey	Bellemore			Henry Ford Health	Detroit, MI	Lab Staff/Lab Manager	CAS, WHEALS
Jocelyn M.	Biagini		PhD	Division of Asthma Research, Cincinn	Cincinnati, OH	Coinvestigator	CCAAPS
Dean	Billheimer		PhD	University of Arizona	Tucson, AZ	Biostatistician	IIS
Alex	Binder			University of Wisconsin-Madison	Madison, WI	Data Scientist	CREW Informatics Team
Geraldine	Birg			Henry Ford Health	Detroit, MI		CAS
Rashida	Blackwood			Icahn School of Medicine at Mount S	New York, NY	Lab personnel	CCCEH, URECA
Shirley	Blocki			Henry Ford Health	Detroit, MI		CAS
Gordon	Bloomberg			Washington University School of Me	St Louis, MO	Coinvestigator	URECA
Kevin	Bobbitt			Henry Ford Healths	Detroit, MI	Lab Staff (MAAP)/Coin	WHEALS
Yury	Bochkov			University of Wisconsin-Madison	Madison, WI	Coinvestigator	COAST
Karen	Bourgeois			Henry Ford Health	Detroit, MI		WHEALS
Homer A.	Boushey		MD	University of California	San Francisco, CA	Investigator	WHEALS
Molly	Brausch-Bradner			Cincinnati's Children Hospital Medica	Cincinnati, OH	Clinical Research Coor	CCAAPS
Rebecca	Brockman-Schneider			University of Wisconsin-Madison	Madison, WI	Researcher	COAST
Richard	Budrevich			Rho, Inc.	Durham, NC	Senior Software Develo	URECA
Emily	Bull			Boston University School of Medicine	Boston, MA	Research assistant	URECA
Jeffrey W.	Burkle		BS	Cincinnati's Children Hospital	Cincinnati, OH	Data Manager	CCAAPS
Thomas	Callaci			University of Wisconsin-Madison Sch	Madison, WI	Honest Broker	CREW Informatics Team
Janice	Campbell			Henry Ford Health	Detroit, MI		WHEALS
Kirsten	Carlson-Dakes			University of Wisconsin-Madison	Madison, WI		COAST
Tara F.	Carr		MD	University of Arizona	Tucson, AZ	Investigator	IIS
Andrea	Cassidy-Bushrow			Henry Ford Healths	Detroit, MI	Coinvestitgator/Epiden	WHEALS
Toni	Choiminski			Henry Ford Health	Detroit, MI		WHEALS
Jo Ann	Colas			Rho, Inc.	Durham, NC	Statistical Programmer	CREW Coordinating Center
Michelle	Cootauco			Johns Hopkins University School of M	Baltimore, MD	Lead Coordinator	URECA
Gina	Crisafi			Department of Pediatrics, University	Madison, WI	Program Manager	CREW Administrative Center
Amanda	Cyrus			Henry Ford Health	Detroit, MI	Epi Coodinator	CAS, WHEALS
Douglas	DaSilva			University of Wisconsin-Madison	Madison, WI		COAST
Brent	Davidson			Henry Ford Health	Detroit, MI	Coinvestigator/OB (CA	CAS, WHEALS
Qian	Di		PhD	Department of Environmental Health	Cambridge, MA; Beijing, Ch	Contributor	CREW Geospatial Science Center
Amy	Eapen			Henry Ford Healths	Detroit, MI	Investigator	WHEALS

Supplemental Online Content: Nonauthor Collaborators
*First name, last name, and suffix (if applicable) are required and will appear in PubMed.

***First Name and Middle Initial(s)**	***Last Name**	***Suffix (eg, Jr, III)**	Academic Degrees	Institution	Location (city, state/province, country)	Role or Contribution, eg, chair, principal investigator	Group (if more than 1 Group listed in the byline) and/or Subgroup (eg, Steering Committee)
Heidi	Erickson		BSN, RN	University of Arizona	Tucson, AZ	Pulmonary Research M	IIS
Rachel	Ethridge			Rho, Inc.	Durham, NC	Research Associate	CREW Coordinating Center
Leanna	Farnham			The Channing Division of Network M	Boston, MA	Research Laboratory M	EHAAS
Angela	Freie			Washington University School of Me	St Louis, MO	Study Coordinator	URECA
Lisa	Gagalis			National Institute of Allergy and Infe	Bethesda, MD		URECA
Ronald E.	Gangnon			University of Wisconsin-Madison	Madison, WI	Biostatistician/Data Ma	COAST, CREW Geospatial Science Center
Peter	Gergen			National Institute of Allergy and Infe	Bethesda, MD		URECA
Nicole	Gonzalez			Boston University School of Medicine	Boston, MA	Study Coordinator	URECA
Katherine	Graham McNeil			Henry Ford Health	Detroit, MI	Lead Coordinator (MA	CAS, WHEALS
Lisa	Gress			University of Wisconsin-Madison	Madison, WI	Research Data Analyst	CREW Geospatial Science Center, CREW Informatics Team
Kristine	Grindle			Department of Pediatrics, University	Madison, WI	Lab personnel	COAST
Brian	Hallmark		PhD	University of Arizona	Tucson, AZ	Biostatistician	IIS
Marilyn	Halonen		PhD	Department of Pharmacology, Univer	Tucson, AZ	Investigator	IIS
Jaime	Hart		ScD	Department of Environmental Health	Boston, MA	Contributor	CREW Geospatial Science Center
Suzanne	Havstad			Henry Ford Health	Detroit, MI	Biostatistician, retired	CAS, WHEALS
Lori	Hoepner		MPH, DrPH	Columbia Center for Children's Envir	New York, NY	Data Manager/Program	CCCEH
Daniel J.	Jackson		MD	Department of Pediatrics, University	Madison, WI	Principal Investigator	COAST
Peter	James		ScD, MPH	Department of Environmental Health	Boston, MA	Contributor	CREW Geospatial Science Center
Seth	Jenkins		BS	Cincinnati’s Children Hospital Medica	Cincinnati, OH	Research assistant	CCAAPS
Molly	Johnson			Rho, Inc.	Durham, NC	Senior Biostatistician	URECA
Kyra	Jones			Henry Ford Health	Detroit, MI	Lead Coordinator/Data	CAS, WHEALS
Paul	Jones III	III		Johns Hopkins University School of M	Baltimore, MD	Study Coordinator	URECA
Jorja	Kahn		BS	Brigham and Women's Hospital; Harv	Boston, MA	Research Assistant	CREW Geospatial Science Center, EHAAS
Meyer	Kattan			Columbia University Medical Center	New York, NY	Principal Investigator	URECA
Haejin	Kim			Henry Ford Health	Detroit, MI	Coinvestigator/Allergis	WHEALS
Mark	Kolar			Henry Ford Health	Detroit, MI	Research assistant	CAS
Laura	Ladick			University of Wisconsin-Madison	Madison, WI	Informatics Project Ma	CREW Informatics Team
Carin	Lamm		MD	Columbia University Medical Center	New York, NY	Investigator	URECA
Kristine	Lee			University of Wisconsin-Madison	Madison, WI	Biostatistician	CREW Geospatial Science Center
Stephanie	Leimenstoll			Johns Hopkins University School of M	Baltimore, MD	Lead Coordinator	URECA
Robert F.	Lemanske, Jr.		MD	University of Wisconsin-Madison	Madison, WI	Principal Investigator	COAST
Grace K.	LeMasters			Cincinnati’s Children Hospital	Cincinnati, OH	Investigator	CCAAPS
Albert M.	Levin			Henry Ford Healths	Detroit, MI	Genetic Epidemiologist	WHEALS
Chris	Lim			University of Arizona	Tucson, AZ	Investigator	CREW Geospatial Science Center
Stephanie	Lovinsky-Desir			Columbia University Medical Center	New York, NY	Investigator	URECA
Ana	Manuelian			Boston University School of Medicine	Boston, MA	Research assistant	URECA
Lisa J.	Martin		PhD	Department of Pediatrics, University	Cincinnati, OH	Biostatistician/Coinves	CCAAPS
Jomol	Matthew			University of Wisconsin-Madison Sch	Madison, WI	Informatics Lead	CREW Informatics Team
Judith	McCullough			Henry Ford Health	Detroit, MI		CAS

*First name, last name, and suffix (if applicable) are required and will appear in PubMed.

*First Name and Middle Initial(s)	*Last Name	*Suffix (eg, Jr, III)	Academic Degrees	Institution	Location (city, state/province, country)	Role or Contribution, eg, chair, principal investigator	Group (if more than 1 Group listed in the byline) and/or Subgroup (eg, Steering Committee)				
Miranda	McDonald Stahl			Brigham and Women’s Hospital, Harv	Boston, MA	Research Assistant	EHAAS				
Lance	Mikus			University of Wisconsin-Madison	Madison, WI		COAST				
Wayne J.	Morgan		MD	University of Arizona	Tucson, AZ	Investigator	IIS				
Mariné	Nalbandyan			University of Wisconsin-Madison Sch	Madison, WI	Data Scientist	CREW Informatics Team				
George T.	O’Connor			Boston University School of Medicine	Boston, MA	Principal Investigator	URECA				
Sharon	O’Toole			Brigham and Women’s Hospital, Harv	Boston, MA	Project Manager	CREW Geospatial Science Center, EHAAS				
Tressa	Pappas			Department of Pediatrics, University	Madison, WI	Lab personnel	COAST				
Elsie	Parmar		BS	Cincinnati’s Children Hospital Medica	Cincinnati, OH	Clinical Research Coord	CCAAPS				
Brenda	Patterson			Washington University School of Med	St Louis, MO	Study Nurse	URECA				
Kelly	Penke			Rho, Inc.	Durham, NC	Project Manager	CREW Coordinating Center				
Frederica	Perera		DrPH, PhD	Columbia Center for Children's Envirc	New York, NY	Investigator	CCCEH				
Matthew	Perzanowski		PhD	Columbia Center for Children's Envirc	New York, NY	Investigator	CCCEH				
Edward	Peterson			Henry Ford Health	Detroit, MI		CAS				
Marcela	Pierce		MPH	Columbia University Medical Center	New York, NY	Study Coordinator	URECA				
Victoria	Rajamanickam			University of Wisconsin-Madison	Madison, WI	REDCAap System Engin	COAST				
Judyth	Ramirez			Columbia University Medical Center	New York, NY	Lead Coordinator	CCCEH				
Kimberly	Ray			Washington University School of Med	St Louis, MO	Study Coordinator	URECA				
Chris M	Reyes			University of Wisconsin-Madison	Madison, WI	Publications Director	CREW Administrative Center				
Kylie	Riley			Columbia University Medical Center	New York, NY	Data Manager/Study C	CCCEH				
Katherine	Rivera-Spoljaric			Washington University School of Med	St Louis, MO	Principal Investigator/C	URECA				
Kathleen	Roberg			University of Wisconsin-Madison	Madison, WI		COAST				
Olivia	Salamon			Rho, Inc.	Durham, NC	Associate Project Mana	CREW Coordinating Center				
Lisa	Salazar			Department of Pediatrics, University	Madison, WI	Study Coordinator	COAST				
Hugh	Sampson			Icahn School of Medicine at Mount S	New York, NY	Investigator	URECA				
Megan T.	Sandel			Boston University School of Medicine	Boston, MA	Investigator	URECA				
Ruchika	Sangani			Boston University School of Medicine	Boston, MA	Investigator	URECA				
Leena	Sathe		PhD	Brigham and Women's Hospital; Harv	Boston, MA	Lab Manager	EHAAS				
Joel	Schwartz		PhD	Department of Environmental Health	Boston, MA	Investigator	CREW Geospatial Science Center				
Dena	Scott			Johns Hopkins University School of M	Baltimore, MD	Research Assistant	URECA				
Gina	Simpson			Washington University School of Med	St Louis, MO	Study Coordinator	URECA				
Sweta	Singh			University of Wisconsin-Madison	Madison, WI	Data Manager	CREW Informatics Team				
Sweta	Singh			University of Wisconsin-Madison	Madison, WI	Data Manager	CREW Geospatial Science Center, CREW Informatics Team				
Alexandra	Sitarik			Henry Ford Health	Detroit, MI	Biostatistician	CAS, WHEALS				
Ronald	Sorkness			University of Wisconsin-Madison	Madison, WI	Coinvestigator	COAST				
Daniel	Spagna		BS	Cincinnati’s Children Hospital Medica	Cincinnati, OH	Research assistant	CCAAPS				
Amber	Spangenberg			University of Arizona	Tucson, AZ	Lab personnel	IIS				
Rhoda	Sperling			Icahn School of Medicine at Mount S	New York, NY	Investigator	URECA				
David	Spies			University of Arizona	Tucson, AZ	Data Manager	IIS				
Debra A.	Stern		MS	Asthma and Airway Disease Research	Tucson, AZ	Lead Coordinator	IIS				

Supplemental Online Content: Nonauthor Collaborators
*First name, last name, and suffix (if applicable) are required and will appear in PubMed.

***First Name and Middle Initial(s)**	***Last Name**	***Suffix (eg, Jr, III)**	Academic Degrees	Institution	Location (city, state/province, country)	Role or Contribution, eg, chair, principal investigator	Group (if more than 1 Group listed in the byline) and/or Subgroup (eg, Steering Committee)				
Jeff	Stokes			Washington University School of Med	St Louis, MO	Coinvestigator	URECA				
Cathey	Strauchman Boyer			Henry Ford Health	Detroit, MI		CAS				
Caitlin	Suddueth			Rho, Inc.	Durham, NC	Project Manager	CREW Coordinating Center				
William	Taylor			Rho, Inc.	Durham, NC	Clinical Data Manager	URECA				
Christopher	Tisler			Department of Pediatrics, University	Madison, WI	Lab Manager	COAST				
Alkis	Togias			National Institute of Allergy and Infec	Bethesda, MD		URECA				
Audrey	Urquhart			Henry Ford Health	Detroit, MI	Data Manager (CAS, M.	CAS, WHEALS				
Anthony	Wahlman		BA	Henry Ford Health	Detroit, MI	Research Programmer	CAS, WHEALS				
Ganesa	Wegienka			Henry Ford Health	Detroit, MI	Coinvestigator (CAS, M	CAS, WHEALS				
Karen	Wells			Henry Ford Health	Detroit, MI		CAS				
L. Keoki	Williams			Henry Ford Health	Detroit, MI		WHEALS				
Seth	Wilson			Brigham and Women's Hospital; Harv	Boston, MA	TRA	EHAAS				
Robert A.	Wood		MD	Johns Hopkins University School of M	Baltimore, MD	Principal Investigator	URECA				
Kimberley	Woodcroft			Henry Ford Health	Detroit, MI	Lab Director/Molecular	CAS, WHEALS				
Melissa	Yaeger			University of Wisconsin-Madison	Madison, WI	Program Manager	CREW Administrative Center				
Perri	Yaniv			Columbia University	New York, NY	Lead Coordinator	URECA				
Jeffrey D.	Yanosky			Institutes of Energy and the Environn	University Park, PA	Investigator	CREW Geospatial Science Center				
Qiong	Zhang			Henry Ford Healths	Detroit, MI	Biostatistician	CREW Geospatial Science Center				
Shirley	Zhang			Henry Ford Healths	Detroit, MI	Senior Research Progra	CAS, WHEALS				
