# Supplementary material for: Early-Life Exposure to Air Pollution and Childhood Asthma Cumulative Incidence in the ECHO CREW Consortium
Source: JAMA Netw Open. 2024 Feb 28;7(2):e240535. doi: 10.1001/jamanetworkopen.2024.0535 (PMC10902721; doi:10.1001/jamanetworkopen.2024.0535)
Supplement: Supplement 3. — Data Sharing Statement [file jamanetwopen-e240535-s003.pdf]

Data Sharing Statement

Zanobetti. Early-Life Exposure to Air Pollution and Childhood Asthma Cumulative Incidence in the ECHO/CREW Consortium. *JAMA Netw Open*. Published February 28, 2024. doi:10.1001/jamanetworkopen.2024.0535

Data
Data available: No

Additional Information
Explanation for why data not available: The data are not publicly available due to linkage to potentially identifying and sensitive patient information of participants in three National Institutes of Health (NIH) Environmental Influences on Child Health Outcomes (ECHO)'s Children's Respiratory and Environmental Workgroup (CREW) consortium birth cohorts. All cohorts are governed by DUA and institutional IRBs. Access to confidential ECHO/CREW data requires written authorization from the ECHO/CREW study sponsor, the ECHO/CREW Program, and a data request submitted to the ECHO/CREW PI.
